# High PRMT5 levels, maintained by KEAP1 inhibition, drive chemoresistance in high-grade serous ovarian cancer

**DOI:** 10.1172/JCI184283

**Published:** 2025-03-17

**Authors:** Harun Ozturk, Fidan Seker-Polat, Neda Abbaszadeh, Yasemin Kingham, Sandra Orsulic, Mazhar Adli

**Affiliations:** 1 Robert Lurie Comprehensive Cancer Center, Department of Obstetrics and Gynecology, Feinberg School of Medicine at Northwestern University, Chicago, Illinois, USA.; 2 Department of Obstetrics and Gynecology, David Geffen School of Medicine, UCLA, Los Angeles, California, USA.

**Keywords:** Cell biology, Genetics, Oncology, Cancer, Drug therapy

## Abstract

Protein arginine methyl transferases (PRMTs) are generally upregulated in cancers. However, the mechanisms leading to this upregulation and its biological consequences are poorly understood. Here, we identify PRMT5, the main symmetric arginine methyltransferase, as a critical driver of chemoresistance in high-grade serous ovarian cancer (HGSOC). PRMT5 levels and its enzymatic activity are induced in a platinum-resistant (Pt-resistant) state at the protein level. To reveal potential regulators of high PRMT5 protein levels, we optimized intracellular immunostaining conditions and performed unbiased CRISPR screening. We identified Kelch-like ECH-associated protein 1 (KEAP1) as a top-scoring negative regulator of PRMT5. Our mechanistic studies show that KEAP1 directly interacted with PRMT5, leading to its ubiquitin-dependent degradation under normal physiological conditions. At the genomic level, ChIP studies showed that elevated PRMT5 directly interacted with the promoters of stress response genes and positively regulated their transcription. Combined PRMT5 inhibition with Pt resulted in synergistic cellular cytotoxicity in vitro and reduced tumor growth in vivo in Pt-resistant patient-derived xenograft tumors. Overall, the findings from this study identify PRMT5 as a critical therapeutic target in Pt-resistant HGSOC cells and reveal the molecular mechanisms that lead to high PRMT5 levels in Pt-treated and chemo-resistant tumors.

## Abstract 2

KEAP1 inhibition elevates PRMT5 levels, driving chemoresistance in HGSOC by regulating stress response genes. PRMT5 inhibition sensitizes resistant tumors to platinum, improving treatment efficacy.

## Introduction

Ovarian cancer (OC) is the deadliest gynecologic cancer and takes the lives of more than 15,000 women each year in the United States alone (1). High-grade serous ovarian carcinomas (HGSOCs), the most aggressive OC subtype, account for 80% of OC-related deaths (1). Given the universal tumor protein P53 (*TP53*) mutations and recurrent alterations in DNA repair pathways, these tumors are initially responsive to platinum-based (Pt-based), DNA-damaging chemotherapy. However, tumors rapidly recur, and most patients (80%) die as a result of Pt-resistant tumors (1, 2). Despite extensive genome-wide genetic (3–5) and epigenetic (4, 6–8) efforts, the critical drivers of Pt resistance are still unknown, and none of the recurrent genetic mutations in Pt-resistant cases (such as cyclin E1 [*CCNE1*] amplification and breast cancer gene 1/2 [*BRCA1/2*] reversion) observed in less than 30% of resistant tumors are clinically targetable. Despite excitement about the immune checkpoint modulators, given their remarkable clinical benefits in certain cancers (9, 10), they have had minimal success in the clinical management of OC (11, 12). Therefore, the first-line treatment of OC still relies on conventional DNA-damaging chemotherapy, with Pt being the backbone of these therapies. Although these tumors are initially sensitive to chemotherapy, because of recurrent mutations in several DNA repair machinery genes, they rapidly evolve into a chemo-resistant state, which ultimately leads to death of the patient (13). Therefore, understanding and targeting the molecular drivers of chemoresistance remains a major challenge in the management and therapeutic interventions for OC.

High-throughput loss-of-function screening is a promising approach to discovering the drivers of chemoresistance and identifying synergistic pathways upon which the resistant cells selectively depend. Such screening may also reveal combinatorial drug targets whose inhibition results in synthetic lethality. Through in vivo CRISPR screening, we previously identified protein arginine methyltransferase 5 (PRMT5) as a synthetically lethal partner of gemcitabine (14), a cytosine analog and backbone of several chemotherapies. Our mechanistic studies, supported by other independent reports, highlighted that PRMT5 is a key component of efficient DNA repair program (14–18). Notably, PRMT5 catalyzes mono- and symmetric dimethylation of arginine residues in various proteins, including histones and transcription factors (19, 20). The human genome encodes 9 PRMTs. Type I PRMTs perform mono and asymmetric dimethylation, whereas type II PRMTs perform mono and symmetric arginine methylation (21). Almost all symmetric arginine methylation is performed by PRMT5 (20). The PRMT5-mediated arginine methylation is implicated in various biological processes, including transcription, RNA splicing, and translation (20, 22).

Notably, PRMT5 expression is upregulated in several cancers. However, what leads to this upregulation and the pathogenic mechanisms downstream of it that contribute to disease initiation, progression, and aggressiveness are poorly understood (23–28). In this study, we investigated how PRMT5 levels and its enzymatic activity are upregulated in HGSOC and whether it plays a crucial role in the acquisition of Pt chemoresistance. Through genetic and pharmacological manipulations, biochemical characterization, and high-throughput functional genomic screening, we revealed the mechanism of why PRMT5 is aberrantly upregulated in HGSOC and studied its role in driving chemoresistance. Our findings revealed that OC cells, upon loss of the *TP53* gene, enhanced the expression of *PMRT5* at the transcriptional level. Notably, PRMT5 protein levels and its enzymatic activity were further induced in Pt-resistant OC cells. Through an unbiased CRISPR gene-KO screening, we identified that high PRMT5 protein levels in chemo-resistant cells were induced by Kelch-like ECH-associated protein 1 (KEAP1) inhibition with Pt-treatment. We found that under normal physiological conditions, KEAP1 directly interacted with PRMT5 and led to its degradation in a ubiquitin-dependent manner. However, cellular stress conditions, such as those induced with Pt treatment, inhibited KEAP1 and resulted in PRMT5 protein stabilization and upregulation at the protein level. Finally, we show that the Pt-resistant cells, which had higher PRMT5 levels, selectively depended on PRMT5 activity to survive chemotherapy. As such, the combinatorial PRMT5 inhibition with carboplatin resulted in synergistic DNA damage accumulation and apoptotic cytotoxicity in vitro, and the combinatorial treatment blocked the growth of otherwise Pt-resistant patient-derived xenograft (PDX) tumors in vivo.

## Results

### High expression of PRMT5 in TP53-mutant tumors is associated with poor prognosis.

**Figure 1:**
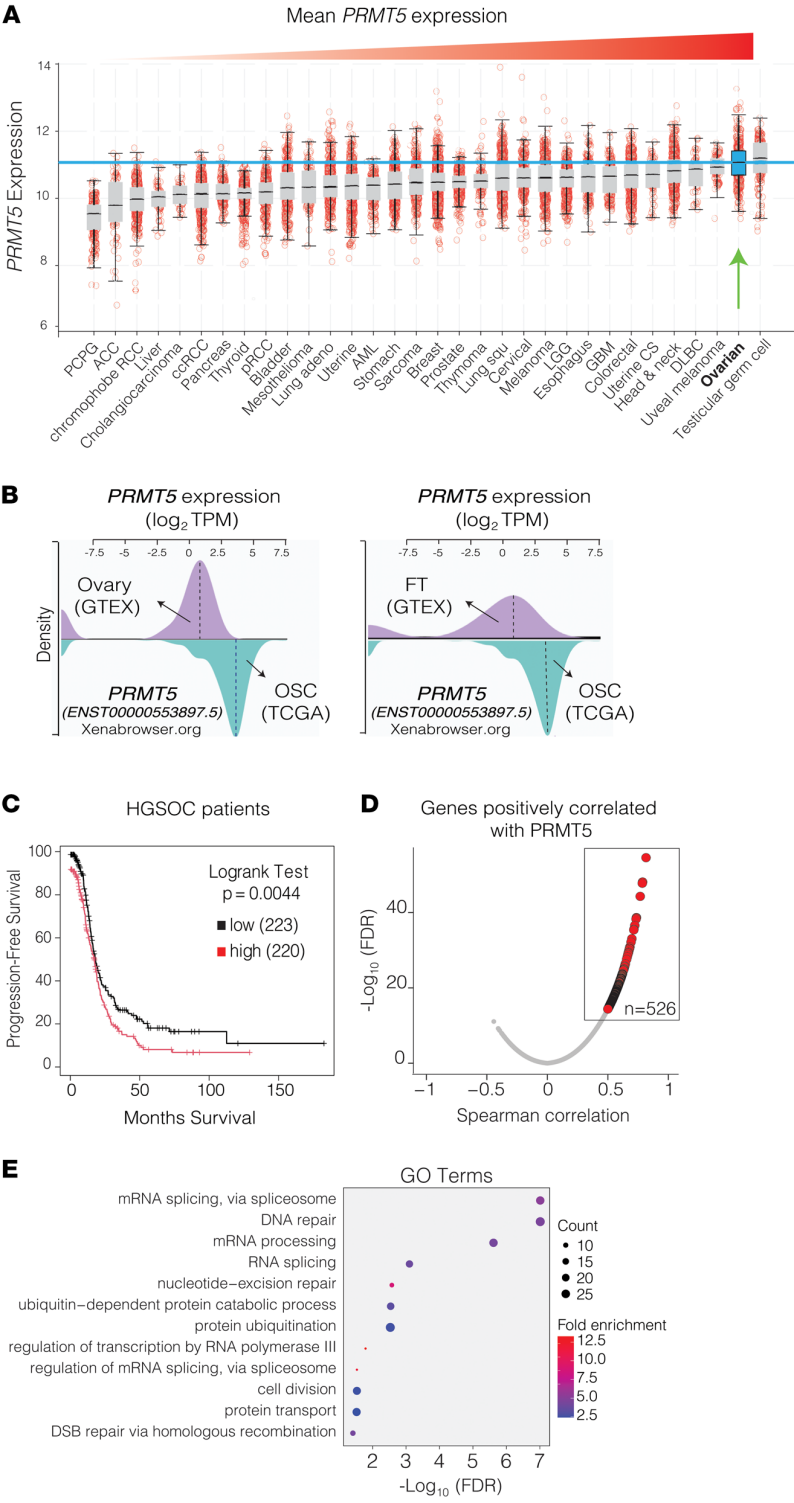
PRMT5 is highly expressed in OC and associated with poor patient survival. (**A**) Box plots display mean *PRMT5* mRNA expression across 32 different human cancer types. The green arrow indicates PRMT5 expression in OC, and the blue line indicates mean PMRT5 expression in OC tumors. (**B**) Density plots compare PRMT5 expression between ovary versus OSC (left) and fallopian tube (FT) versus OSC (right). OSC, ovarian serous cystadenocarcinoma. (**C**) The Kaplan-Meier curve shows progression-free survival between HGSOC patients with tumors expressing low or high levels of PRMT5. The *P* value was determined by the log-rank (Mantel-Cox) test. (**D**) Ranked correlation of genes with PRMT5 expression based on TCGA-HGSOC dataset. Red circles highlight genes whose expression showed a significant positive correlation with PRMT5 expression (*n* = 526, FDR >0.0001, Spearman correlation >0.5). (**E**) Dot plot illustrating the associated GO terms for the genes identified in **D**.

We and others have previously shown that PRMT5 is a key player in DNA repair pathways (14–18). Since DNA repair pathways are frequently misregulated in cancers, we investigated whether PRMT5 is abnormally regulated in specific cancer types and whether its aberrant regulation is associated with specific genetic abnormalities. To this end, we initially studied *PRMT5* expression in more than 1,000 cancer cells in the Cancer Cell Line Encyclopedia (CCLE) database (29). Critically, we identified that *PRMT5* expression was significantly upregulated in *TP53*-mutant cell lines (Supplemental Figure 1A; supplemental material available online with this article; https://doi.org/10.1172/JCI184283DS1). The p53 transcription factor is a crucial regulator of genome stability through its transcriptional control of genes involved in DNA damage repair and apoptosis (30). Importantly, *TP53* is mutated and lost in more than 50% of all cancers and ubiquitously lost in nearly all HGSOC tumors (31). We therefore explored The Cancer Genome Atlas (TCGA) data to study cancer type–specific expression patterns in *PRMT5*. Notably, in line with the CCLE data, we observed that OC, as a whole, had the second-highest expression of *PRMT5* compared with all other cancer types (Figure 1A). Supporting this finding, we observed that *PRTM5* expression was substantially higher in OC tumors than in either normal ovarian epithelial cells or fallopian tube epithelial cells, both of which are believed to be the cell of origin for HGSOC (32–34) (Figure 1B). Importantly, depletion of *Tp53* in murine ID8 cells resulted in significant PRMT5 upregulation at both the mRNA and protein levels (Supplemental Figure 1, B and C), further supporting the idea that *TP53* loss is a critical contributor to high *PRMT5* mRNA levels in HGSOC tumors and potentially contributes to the pathogenesis of this disease. In line with this, we found that patients with high *PRMT5*-expressing tumors had significantly shorter progression-free survival (Figure 1C), demonstrating that PRMT5 levels are a notable prognostic factor and a potential therapeutic vulnerability in HGSOC. To reveal whether *PRMT5* expression is associated with key pathways implicated in disease pathogenesis and clinical outcomes, we investigated genes whose expression is coregulated with *PRMT5* expression in HGSOC tumors. We found that a total of 526 such genes were significantly positively correlated with *PRMT5* expression (Figure 1D; FDR <0.0001, Spearman correlation score >0.5). Importantly, the Database for Annotation, Visualization, and Integrated Discovery (DAVID) GO analysis highlighted that these genes are significantly enriched for DNA repair activity (35, 36) (Figure 1E; FDR <0.0001). Given that the DNA repair pathways are often misregulated in HGSOC at the genetic level (37), these findings indicate that high *PRMT5* expression could be at least one mechanism of how these tumors compensate for the genetic loss of these pathways to become Pt resistant.

### PRMT5 upregulation drives chemoresistance to Pt.

**Figure 2:**
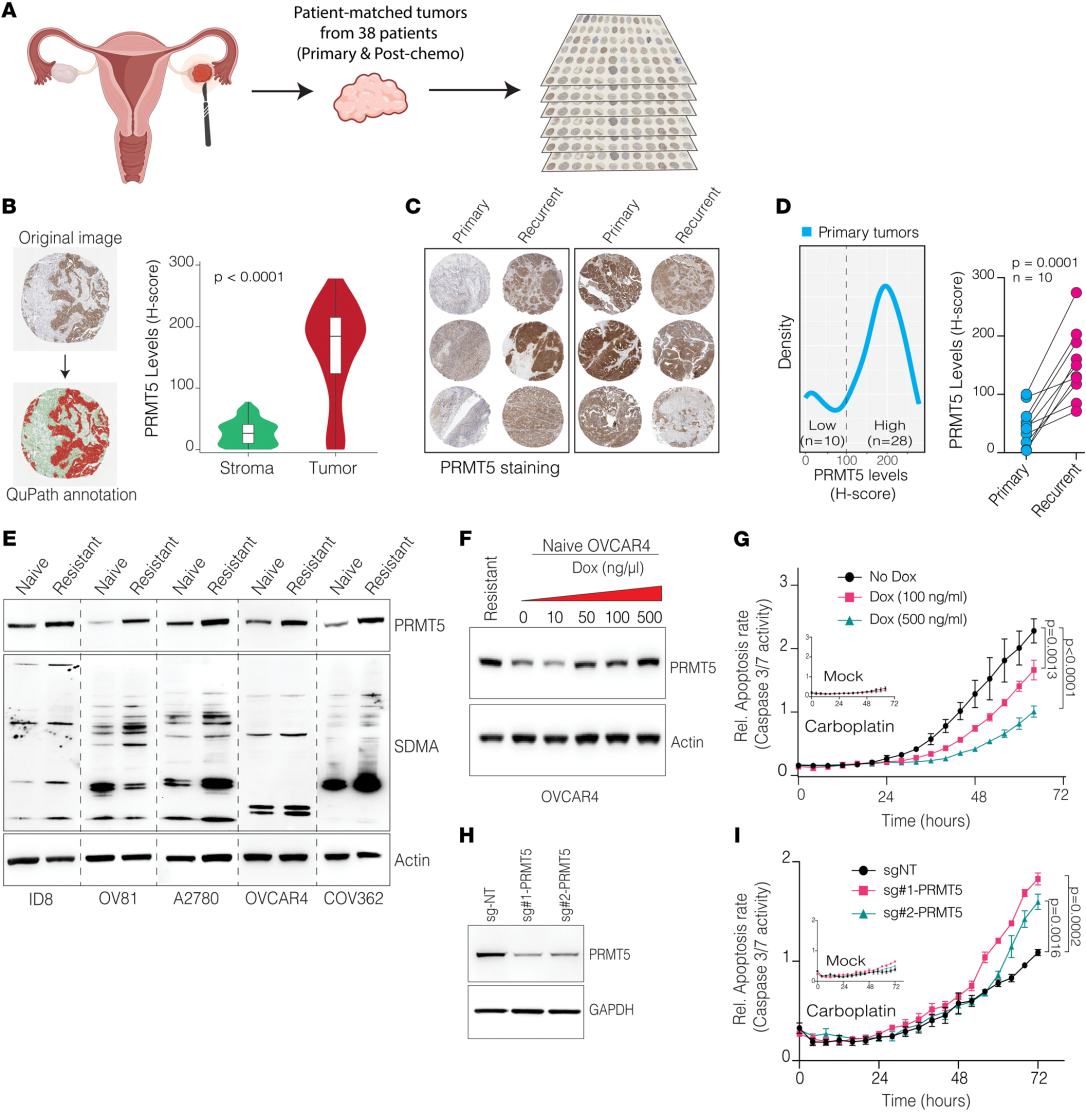
PRMT5 protein levels are further induced in chemotherapy-treated and chemotherapy-resistant HGSOC tumors. (**A**) Schematic displays TMAs used for PRMT5 staining. Figure was created with BioRender.com. (**B**) Representative IHC images show QuPath annotation of tumor stroma and epithelial cells (left), and a violin plot shows PRMT5 staining intensity in stroma versus tumor epithelial cells (right). *P* value was determined by Wilcoxon rank-sum test. Original magnification: ×5. (**C**) Representative IHC images show PRMT5 staining in primary and recurrent tumors. Original magnification: ×5. (**D**) The density plot displays H-scores for PRMT5 staining in primary tumors (left), and the dot plot shows PRMT5 levels (H-score) between primary and recurrent tumors for the primary tumors with low PRMT5 staining (right). *P* values were quantified by 2-tailed, paired Student’s *t* test. (**E**) Western blots show PRMT5 and SDMA levels in chemo-naive and -resistant isogenic cell line pairs. Noncontiguous lanes on different blots have been separated by thin dashed lines. (**F**) Western blots show PRMT5 expression after different doses of Dox induction (72 hours) in chemo-naive OVCAR4 cells. Carboplatin-resistant OVCAR4 cells were used to determine Dox levels for overexpression. (**G**) Line plots show the relative apoptosis rate of OVCAR4 cells treated with the indicated doses of Dox. Cells were subjected to 40 μM carboplatin, and apoptosis (caspase 3/7 activity) was monitored by the IncuCyte live-cell imaging platform for 72 hours. Data are shown as the mean ± SEM (*n* = 3). *P* values were quantified by 1-way ANOVA with Dunnett’s multiple-comparison test. (**H**) Western blots show PRMT5 levels in OVCAR4 cells expressing sgRNAs targeting PRMT5 promoter. (**I**) Line plots show the relative apoptosis rates of OVCAR4 cells expressing the indicated sgRNAs. Cells were treated with 20 μM carboplatin, and apoptosis was monitored over 72 hours using the IncuCyte live-cell imaging platform. Data are shown as the mean ± SEM (*n* = 3). *P* values were determined by 1-way ANOVA with Dunnett’s multiple-comparison test.

The above analysis indicates that OC tumors have high *PRMT5* mRNA expression. We wanted to confirm whether PRMT5 protein levels are also high in chemo-naive HGSOC tumors and whether PRMT5 levels change during tumor recurrence and chemoresistance. To this end, we performed immunohistochemical staining of PRMT5 in well-annotated tissue microarrays (TMAs) composed of patient-matched primary and recurrent HGSOC (Figure 2A). Using QuPath software (38), we calculated H-scores from 112 tumor samples obtained from 38 patients. In line with the gene expression data, we found that PRMT5 protein levels were significantly higher and almost exclusively restricted to tumor cells compared with stromal cells (Figure 2B). We then studied intertumor heterogeneity of PRMT5 expression across chemo-naive tumors and recurrent post-chemotherapy tumors. Notably, we observed bimodal distribution of PRMT5 levels in chemo-naive tumors, in which some had low and others had high PRMT5 protein levels in primary chemo-naive tumors (Figure 2, C and D). Notably, those tumors with low PRMT5 levels had uniformly and significantly upregulated PRMT5 expression in a post-chemotherapy recurrent state, implying that PRMT5 may be a key determinant of tumor recurrence and chemoresistance (Figure 2D). Some of the HGSOC tumors already had high basal PRMT5 levels. Intriguingly, most of these high PRMT5 tumors were *BRCA1/2* mutant samples (Supplemental Figure 2A), suggesting that PRMT5 may become more vital in *BRCA1/2*-mutant cells.

These findings led to the hypothesis that PRMT5 is an essential determinant of Pt resistance. To test this, we initially studied whether PRMT5 expression is further induced during Pt resistance. We utilized the isogenic cell lines we previously generated to be Pt resistant upon continuous treatment with Pt (6). Remarkably, in all 5 cell lines (ID8, OV81, A2780, OVCAR4, and COV362), we observed substantially higher PRMT5 protein levels in the Pt-resistant cells, whereas *PRMT5* mRNA levels did not change significantly (Figure 2E and Supplemental Figure 2, B and C). More critically, in line with PRMT5 protein levels, we observe substantially higher symmetric arginine dimethylation (SDMA) levels, indicating that the Pt-resistant cells also have higher PRMT5 enzymatic activity (Figure 2E). To directly test our hypothesis that high PRMT5 levels drive chemoresistance, we generated cells in which PRMT5 levels could be titrated with doxycycline (Dox) (Figure 2F). Importantly, induction of PRMT5 in naive cells at levels comparable to those in resistant cells resulted in significantly lower apoptosis in response to carboplatin treatment (Figure 2G), indicating that high PRMT5 expression was sufficient to drive chemoresistance in a dose-dependent manner. To further test this, we also aimed to epigenetically downregulate PRMT5 levels and assess how this would alter the carboplatin response. To this end, we used CRISPR interference (CRISPRi) technology to epigenetically edit the *PRMT5* promoter and deposit repressive H3K9me3 histone modification via targeted recruitment of dCas9-KRAB (39). Using 2 distinct sgRNAs targeting the *PRMT5* promoter, we noted substantial downregulation of PRMT5 protein levels compared with control sgRNA (Figure 2H). Critically, this reduction in PMRT5 expression resulted in a significant increase in apoptotic cell death following carboplatin treatment (Figure 2I). Overall, these findings establish the crucial role of PRMT5 in rendering cells resistant to Pt-based chemotherapy.

### PRMT5 protein levels are controlled by KEAP1.

**Figure 3:**
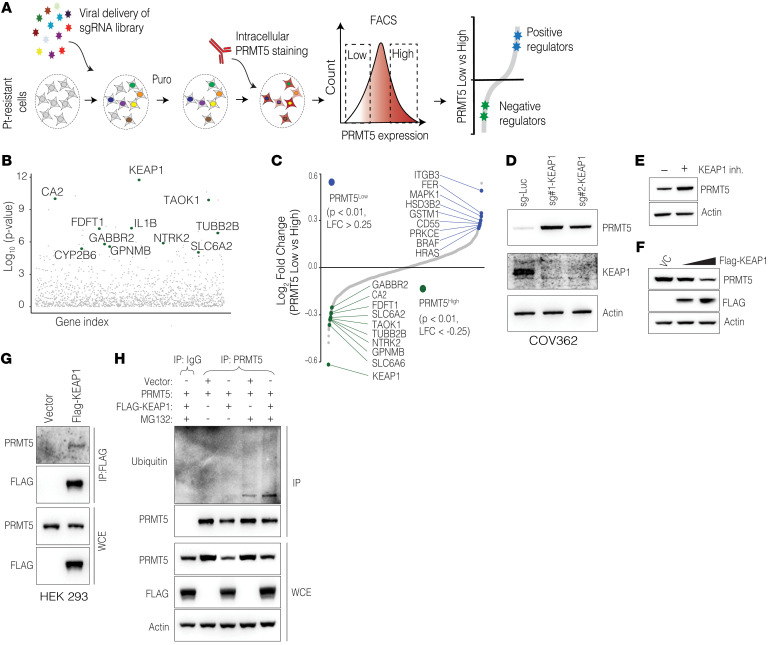
PRMT5 protein levels are regulated by KEAP1-mediated ubiquitination and proteasomal degradation. (**A**) Schematic shows the CRISPR screening strategy used to determine PRMT5 regulators. (**B**) Dot plot shows the significance levels of sgRNAs detected in the PRMT5^hi^ group. (**C**) Dot plot displays the enrichment of sgRNAs. sgRNAs significantly enriched in the PRMT5^lo^ group (*P* < 0.01 and log_2_ FC [LFC] > 0.25) are labeled in blue, and sgRNAs significantly enriched in the PRMT5^hi^ group (*P* < 0.01 and LFC < –0.25) are labeled in green. (**D**) Western blots show PRMT5, KEAP, and actin protein levels in KEAP1-depleted cells. (**E**) Western blots show PRMT5 and actin protein levels between control and KEAP1 inhibitor–treated cells (bardoxolone, 2 μM for 72 hours). (**F**) Western blot shows PRMT5 levels upon 2 different amounts of KEAP1 overexpression (250 and 1,000 ng) in HEK 293 cells. (**G**) Western blot shows co-IP results for the KEAP1-PRMT5 interaction. FLAG-tagged KEAP1 was overexpressed and immunoprecipitated using an anti-FLAG antibody. (**H**) Western blot shows IP results for PRMT5 ubiquitination. KEAP1, or an empty vector, was coexpressed along with PRMT5. Cells were also treated overnight with DMSO or MG132. PRMT5 was pulled down using an anti-PRMT5 antibody, with IgG used as a control, followed by Western blotting with the indicated antibodies. WCE, whole-cell extract.

We next investigated the molecular drivers of high *PRMT5* expression in OC. Our preliminary findings (Supplemental Figure 1, A–C) suggested that high *PRMT5* mRNA levels were likely being driven by the loss of *TP53*. However, the molecular mechanism behind higher PRMT5 protein levels in Pt-resistant cells is not known. Our data suggest that the higher PRMT5 protein levels in resistant cells were not due to higher mRNA expression (Supplemental Figure 2C). We therefore aimed to identify potential drivers of high PRMT5 levels in chemo-resistant cells. To this end, we performed CRISPR gene-KO screening using a custom-designed “druggable” sgRNA library targeting approximately 1,400 proteins with existing small-molecule inhibitors (Figure 3A). To be able to do this, we initially optimized immunostaining conditions that enabled us to quantitatively and differentially measure intracellular PRMT5 levels in naive and resistant cells with flow cytometry (Supplemental Figure 3A). Upon optimizing the PRMT5 staining conditions, we then virally delivered the sgRNA library at a MOI of 0.25 in Cas9-expressing, Pt-resistant COV362 cells. After 2 weeks of sgRNA library expression, we sorted cells into PRMT5^hi^ (top 20%) and PRMT5^lo^ (bottom 20%) populations and quantified the relative abundance of sgRNAs in these 2 populations using house and published methods (40). Importantly, this unbiased screening identified several hits that positively regulate PRMT5 expression, including oncoproteins such as HRAS and BRAF, whereas tumor suppressors like KEAP1 were identified as top-scoring hits that negatively regulate PMRT5 levels (Figure 3, B and C). Crucially, control sgRNAs showed no significant enrichment in either group, confirming the reliability of our screening approach (Supplemental Figure 3, B and C). We designed independent sgRNAs to validate the top hits that up- or downregulate PRMT5 levels. In line with the screening data, we found that depletion of *FER* or *ITGB3* substantially downregulated PRMT5 levels (Supplemental Figure 3, D and E), demonstrating the robustness of our screening. Among the genes that resulted in higher PRMT5 expression, *KEAP1* was the most significant (Figure 3, B and C). KEAP1 is an adaptor protein that brings together its target proteins, such as nuclear factor erythroid 2-related factor 2 (NRF2), with the ubiquitin machinery to facilitate ubiquitin-dependent proteasomal degradation of the target proteins (41, 42). We initially validated that depletion of *KEAP1* at the genetic level with 2 separate sgRNAs resulted in substantial upregulation of PRMT5 protein levels (Figure 3D). As expected, *KEAP1* downregulation also led to an increase in NRF2 levels, in line with the pivotal role of KEAP1 in degrading NRF2 (43) (Supplemental Figure 3F).

Interestingly, we did not observe higher *PRMT5* mRNA levels when *KEAP1* was depleted (Supplemental Figure 3G), suggesting that higher PRMT5 levels upon *KEAP1* depletion were not transcriptionally controlled. We therefore tested the hypothesis that KEAP1 controls PRMT5 levels at the protein level. We used bardoxolone, a pharmacological inhibitor of the KEAP1/NRF2 axis, to further validate these findings. Notably, in line with the genetic depletion of *KEAP1*, we found that its pharmacological inhibition (as evidenced by significantly increased expression of the NRF2 target gene *NQO1*; Supplemental Figure 3H) led to a substantial increase in PRMT5 protein levels (Figure 3E) but did not affect its mRNA levels (Supplemental Figure 3H), indicating that functional KEAP1 was a negative regulator of PRMT5 protein stability. Supporting this observation, we found that exogenous expression of KEAP1 led to notable downregulation of PRMT5 (Figure 3F) in an independent cell line (HEK 293), confirming that KEAP1 was a regulator of PRMT5 protein levels.

Next, we tested whether KEAP1 directly targets PRMT5 protein stability via ubiquitin-mediated degradation. We first checked if PRMT5 physically interacts with KEAP1. We observed strong co-IP of PRMT5 with exogenously expressed KEAP1, indicating that KEAP1 directly interacted with PRMT5 (Figure 3G). To test whether this interaction results in the ubiquitination of PRMT5, we induced exogenous expression of PRMT5 with or without KEAP1. We included MG132, a potent proteasome inhibitor, to prevent proteasomal degradation of PRMT5 and enhance the detection of ubiquitinated PRMT5. Notably, in whole-cell extracts, we observed that KEAP1 overexpression reduced PRMT5 expression, and MG132 treatment partially rescued this degradation, indicating that proteasomal degradation was involved (Figure 3H). Consistent with this, the immunoprecipitated samples revealed a distinct ubiquitin band along with a faint smear, suggesting both mono- and polyubiquitination of PRMT5. This effect was particularly prominent in samples treated with MG132, indicating that ubiquitinated PRMT5 underwent proteasomal degradation. Notably, the intensity of the ubiquitinated PRMT5 markedly increased upon KEAP1 expression (Figure 3H), demonstrating that PRMT5 was ubiquitinated in a KEAP1-dependent fashion. These findings support the hypothesis that KEAP1 regulates PRMT5 protein levels by ubiquitination-dependent proteasomal degradation.

### PRMT5 binds to the promoters and positively regulates stress-response gene expression.

**Figure 4:**
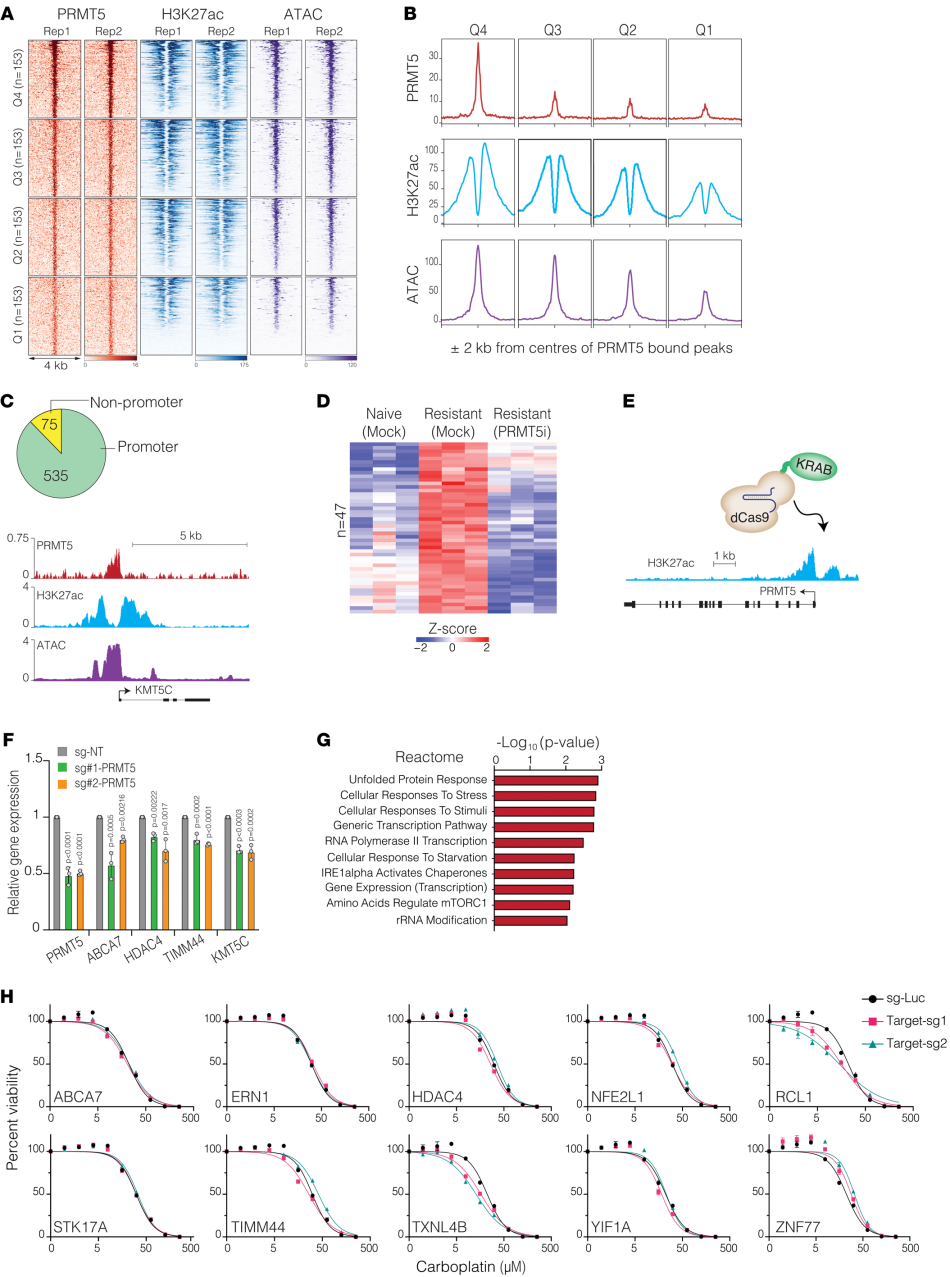
PRMT5 binds to the promoters of stress response genes in Pt-resistant cells. (**A** and **B**) The (**A**) heatmap and (**B**) line plot display PRMT5, H3K27ac ChIP-Seq, and ATAC-Seq signals at the PRMT5-bound regions. Quartiles were created using PRMT5 binding intensity. (**C**) Pie chart shows the number of promoter and nonpromoter-annotated PRMT5-bound regions (top). Genomic tracks representing the *KMT5C* locus (bottom). (**D**) Heatmap shows the expression levels of PRMT5-bound genes in naive, Pt-resistant, and PRMT5i-treated Pt-resistant cells. Only the differentially expressed genes (FDR < 0.05, compared with Pt-resistant cells) are shown. (**E**) Schematic illustrates the dCas9-KRAB system targeting the PRMT5 locus. (**F**) Bar plots display mRNA expression levels of the indicated genes in cells expressing either a control sgRNA or an sgRNA targeting the PRMT5 promoter. Data are presented as the mean ± SD (*n* = 3). *P* values were determined by 1-way ANOVA with Dunnett’s multiple-comparison test. (**G**) Bar plot shows reactome terms associated with the genes shown in **D**. (**H**) Line plots show cell viability results after 6 days of carboplatin treatment in cells expressing the control (luciferase-targeting) and gene-specific sgRNAs. sg-Luc, single-guide luciferase.

As a main symmetric arginine methyl transferase, PMRT5 regulates numerous target proteins, including transcription factors (TFs) and histone proteins, thereby regulating chromatin architecture and gene expression programs (21, 22). In the setting of OC, which genes are directly controlled by PRMT5 and whether PRMT5 positively or negatively regulates these targets are not well understood. Furthermore, there have been contradicting reports about the functional role of the PRMT5-mediated histone arginine methylation in gene transcription. For example, although symmetric dimethylation on histone H4R3 and H3R8 is generally considered to be a repressive epigenetic mark (21), H3R8 methylation also antagonizes the deposition of the repressive H3K27me3 mark by PRC2, thereby contributing to the gene activation (44). To reveal the genomic targets of PRMT5, we mapped PRMT5 binding sites by ChIP-Seq in Pt-resistant cells. Furthermore, to understand whether PRMT5 positively or negatively regulates these targets, we integrated these bindings sites with the chromatin accessibility data by performing the assay for transposase-accessible chromatin with high-throughput sequencing (ATAC-Seq) (45) and the ChIP-Seq map of H3K27ac, an established epigenetic mark of active enhancers and promoters (46, 47). We identified approximately 700 robust PRMT5 binding sites (ChIP-Seq peaks) in the genome. Critically, the vast majority of these binding sites are in the gene promoters (Figure 4, A–C). To understand how PRMT5 binding alters chromatin accessibility and genomic activity, we ranked these sites according to the PRMT5 peak intensity and integrated this with ATAC-Seq and H3K27ac ChIP-Seq signal intensity. Notably, the PRMT5 chromatin binding intensity positively correlated with the chromatin accessibility levels and the active H3K27ac mark intensity (Figure 4B), indicating that the binding of PRMT5 to these promoters results in higher genomic activity and positively regulates these promoters. Next, we wanted to identify a set of genes directly controlled by PRMT5 that may also contribute to chemoresistance in Pt-resistant cells. To this end, we focused on genes upregulated explicitly in resistant cells and those directly dependent on PRMT5 enzymatic activity. Therefore, we integrated gene expression programs of naive, Pt-resistant, and Pt-resistant cells treated with a small-molecule inhibitor of PRMT5 (EPZ015666). This prioritization strategy identified 47 genes whose expression is positively and directly regulated by PRMT5 in Pt-resistant cells (Figure 4D). To confirm that these genes are directly regulated by PRMT5, we induced downregulation of *PRMT5* expression in Pt-resistant OVCAR4 cells using the dCas9-KRAB system with a previously tested set of sgRNAs targeting the *PRMT5* promoter (Figure 4E). This approach achieved a 50% reduction in *PRMT5* expression with both sgRNAs (Figure 4F). Following this downregulation, the expression of PRMT5 target genes also decreased to varying extents (Figure 4F), supporting the idea that PRMT5 directly regulates their expression. Additionally, we validated PRMT5 enrichment at the promoter regions of selected genes using ChIP–quantitative PCR (ChIP-qPCR) (Supplemental Figure 4A). Furthermore, PRMT5 target genes were upregulated in *KEAP1*-KO cells (Supplemental Figure 4B), suggesting that increased PRMT5 levels positively regulated their expression. Notably, the gene ontology (GO) analysis indicated that these genes are highly enriched in unfolded protein response, stress response, and overall transcriptional activity (Figure 4G), indicating that PRMT5 contributes to chemoresistance by positively regulating overall cellular stress pathways in Pt-resistant cells. To test whether the depletion of any of these genes is sufficient to make resistant cells sensitive to carboplatin, we knocked out the selected PRMT5 gene targets with 2 different sgRNAs. Notably, only two (*RCL1* and *TXNL4B*) of the top-10 most promising PRMT5 target genes (*ABCA7*, *ERN1*, *HDAC4*, *NFE2L1*, *RCL1*, *STK17A*, *TIMM44*, *TXNL4B*, *YIF1A*, and *ZNF77*) partially rendered these cells sensitive to Pt (Figure 4H). In addition to PRMT5 target genes, we also generated an *NRF2*-KO cell line to test whether its upregulation due to *KEAP1* depletion is causal to chemoresistance. However, *NRF2*-KO cells did not exhibit any significant change in carboplatin-induced apoptotic cell death (Supplemental Figure 4, C and D). These findings support the hypothesis that the overall transcriptional and enzymatic activity of PRMT5, rather than a specific PRMT5 target gene or NRF2, is required to achieve the maximal Pt resistance phenotype.

### Enzymatic inhibition of PRMT5 results in Pt sensitization and synergistic cytotoxicity.

**Figure 5:**
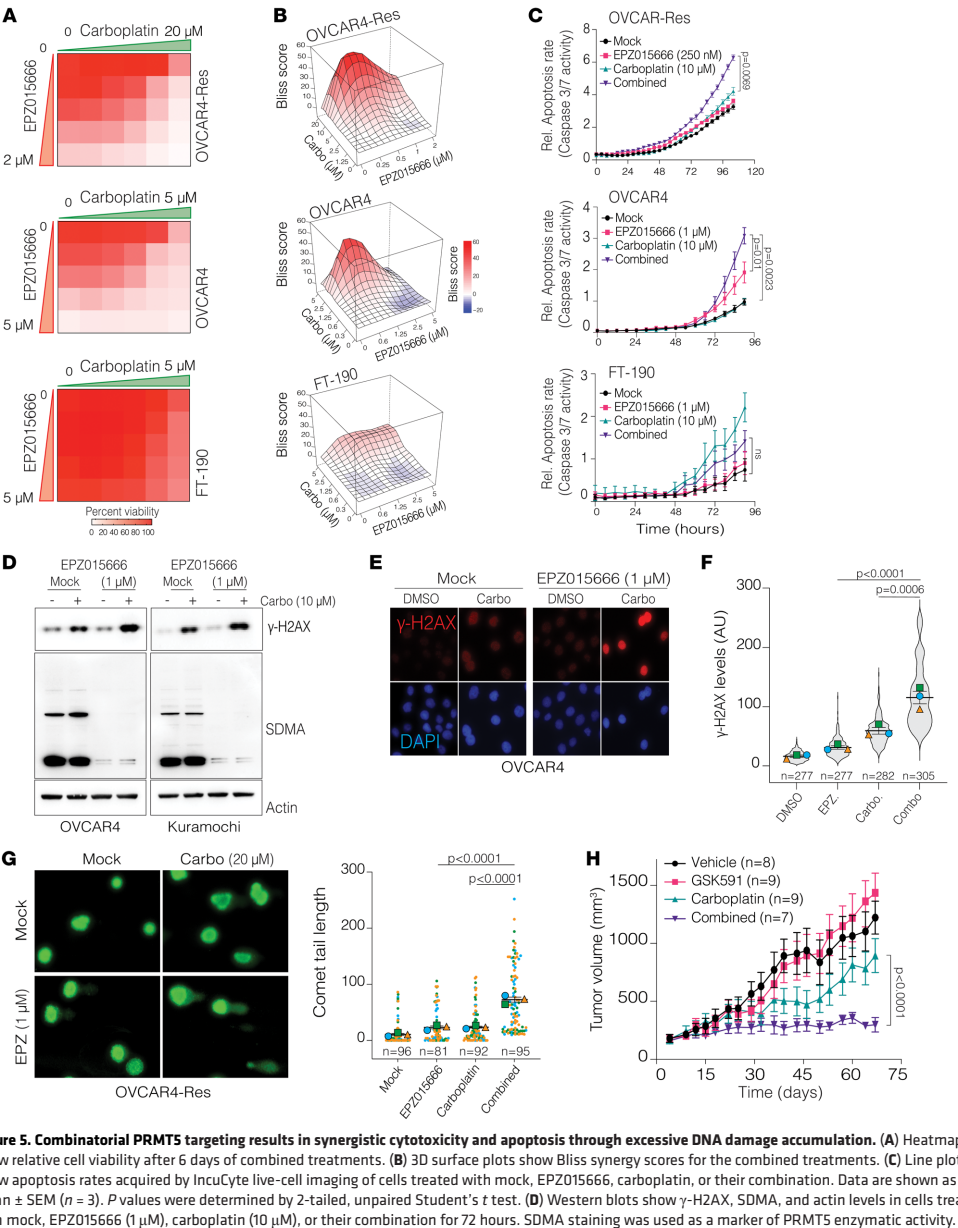
Combinatorial PRMT5 targeting results in synergistic cytotoxicity and apoptosis through excessive DNA damage accumulation. (**A**) Heatmaps show relative cell viability after 6 days of combined treatments. (**B**) 3D surface plots show Bliss synergy scores for the combined treatments. (**C**) Line plots show apoptosis rates acquired by IncuCyte live-cell imaging of cells treated with mock, EPZ015666, carboplatin, or their combination. Data are shown as the mean ± SEM (*n* = 3). *P* values were determined by 2-tailed, unpaired Student’s *t* test. (**D**) Western blots show γ-H2AX, SDMA, and actin levels in cells treated with mock, EPZ015666 (1 μM), carboplatin (10 μM), or their combination for 72 hours. SDMA staining was used as a marker of PRMT5 enzymatic activity. (**E**) IF images show γ-H2AX staining in cells treated with mock, 1 μM EPZ015666, 10 μM carboplatin, or their combination for 72 hours (original magnification, ×40). (**F**) Super plot shows the staining intensity calculated in **E**. Data are shown as the mean ± SEM (*n* = 3). *P* values were determined by 1-way ANOVA with Dunnett’s multiple-comparison test. (**G**) Representative images show individual cell nuclei of carboplatin-resistant OVCAR4 cells treated with mock, 1 μM EPZ015666 (EPZ), 20 μM carboplatin, and their combination for 3 days (left) (original magnification, ×40). Comet tail lengths were quantified and plotted (right). Data are shown as the mean ± SEM (*n* = 3). *P* values were quantified by 1-way ANOVA with Dunnett’s multiple-comparison test. (**H**) Line plot shows tumor growth upon treatment with vehicle GSK591 (50 mg/kg), carboplatin (10 mg/kg), or their combination for approximately 60 days (*n* = ~8 mice/group). Statistical significance was determined by 2-way ANOVA. Carbo, carboplatin; Res, resistant.

The above result led us to test whether global enzymatic inhibition of PRMT5 would result in synergistic cytotoxicity with Pt in naive and Pt-resistant cells. To this end, we performed luminescent cell viability assays to measure the overall cell viability in response to PRMT5 inhibitor treatment alone or in combination with Pt at 5 different doses (6 × 5 = 30 different dose combinations in 3 distinct cell lines). We tested these dose combinations in Pt-naive and Pt-resistant isogenic OVCAR4 cells and normal fallopian tube epithelial cells (FT-190). Critically, we observed a substantial loss of viability at multiple dose combinations in naive and resistant cells but a minimal effect of PRMT5 inhibition on normal epithelial cells (Figure 5A), in line with the fact that these cells expressed markedly lower PRMT5 levels. Because the resistant cells had the highest PRMT5 levels and were Pt resistant, we had to use less PRMT5 inhibitor and substantially higher Pt concentrations to calculate synergy (Figure 5A). We also calculated the bliss synergy score (48) for each dose combination, where a score of greater than 10 indicates strong synergy, and less than –10 indicates antagonistic interaction. Importantly, we observed the highest degree of synergetic cytotoxicity in Pt-resistant cells even though these cells received the lowest PRMT5 inhibitor doses (Figure 5B), indicating that combined PRMT5 inhibition rendered the Pt-resistant cells, which had high PRMT5 levels, sensitive to Pt. Supporting these cell viability data, the IncuCyte live cell imaging measured apoptosis rates (caspase activation) and showed that the combined PRMT5 inhibition resulted in significantly higher apoptosis in Pt-resistant cells despite a lower PRMT5 inhibitor concentration (Figure 5C). We then investigated the potential mechanism of cell death downstream of combination treatment. We observed that only the combined PRMT5 inhibitor and Pt-treated cells had notable overall DNA damage, as indicated by higher γH2AX levels in Western blots (Figure 5D). Notably, we observed a considerable reduction of SDMA, indicating the effective inhibition of PRMT5 enzymatic activity (Figure 5D). We validated these global protein levels with single-cell-level immunofluorescence imaging (Figure 5, E and F). To better understand if PRMT5 inhibition results in synergistic DNA damage accumulation, we performed a comet assay that directly measured fragmented levels of DNA at the single-cell level. Critically, we observed longer comet tails in the combined treatment, indicating significantly higher levels of DNA damage accumulation when PRMT5 was inhibited in Pt-resistant cells (Figure 5G) and thus supporting the findings that PRTM5 contributes to DNA repair activities (14, 16, 17, 49, 50). Additionally, we examined the effect of PRMT5 inhibition on the cell cycle to determine whether it induces cell-cycle arrest. Consistent with our findings, PRMT5-inhibited cells showed a slight accumulation in the S phase (Supplemental Figure 5, A and B), potentially due to increased DNA damage resulting from PRMT5i treatment alone (Figure 5, F and G). To further determine the clinical relevance of these findings, we tested the combination in vivo using a carboplatin-resistant PDX model. Notably, the individual drug treatments did not affect tumor growth over time compared with controls. However, the combination treatment reduced tumor growth significantly, indicating that PRMT5 inhibition in combination with carboplatin resulted in synergistic growth inhibition of chemo-resistant human tumors in vivo (Figure 5H). Importantly, we observed no considerable side effects, as all animals had comparable body weights by the end of the approximately 60-day treatment regimen (Supplemental Figure 5C). These findings highlight the therapeutic relevance of PRMT5 inhibition in treating otherwise chemo-resistant HGSOC tumors.

## Discussion

PRMT5 has emerged as a potentially key therapeutic target in multiple cancers (51). Despite being categorized as an essential gene based on the CRISPR viability score in the DepMap cancer cell line data (52), multiple independent reports highlighted that cancer cells have a greater dependence on PRMT5 activity than do nontransformed cells. More critically, specific genetic abnormalities, such as *MTAP* codeletion with the *CDNK2A* tumor suppressor gene, observed in approximately 15% of cancers, create an additional vulnerability to PRMT5 depletion (53). Here, we demonstrate that PRMT5 is a crucial therapeutic target in HGSOC, especially in its chemo-resistant form. Our findings shed crucial light on why these tumors and their Pt-resistant form have substantially higher levels of PRMT5. We show that, among all cancers, PRMT5 was transcriptionally upregulated in HGSOC tumors and that this transcriptional upregulation was likely due to *TP53* mutations and the impairment of homology-directed repair due to the loss of additional genes such as *BRCA1/2* in these tumors. Importantly, by analyzing PRMT5 levels in large panels of TMAs and isogenic HGSOC cell lines, we show that PRMT5 levels were further induced in the Pt-resistant state.

Notably, our data revealed higher PRMT5 levels in *BRCA1/2*-mutant tumors, indicating higher chemoresistance in such tumors. However, the fact that *BRCA1/2*-mutant tumors are typically more sensitive to Pt treatment due to deficiencies in the homology-directed repair (HDR) pathway presents a paradox. Given the established role of PRMT5 in DNA repair mechanisms (14, 16–18, 54), we think such increased PRMT5 expression is a genetic adaptation mechanism to compensate for the loss of HDR deficiency due to *BRCA1/2* mutation. It is tempting to postulate that higher-than-normal PRMT5 levels are required in the cells so that they can continue to proliferate at a faster rate compared with their WT counterparts. Crucially, we observed even higher levels of PRMT5 upon chemotherapy and in chemo-resistant cells. The fact that combinatorial PRMT5 inhibition rendered these cells substantially more sensitive to chemotherapy indicates that cancer cells require even higher levels of PMRT5 to survive chemotherapy and transition to a chemo-resistant state.

Most notably, we found that the induced PRMT5 levels were driven by posttranslational regulation at the protein level. We identified KEAP1 as a direct regulator of PRMT5 protein levels through unbiased CRISPR screening. Importantly, our findings suggest a model in which PRMT5 levels are regulated at the protein level. Under normal conditions, PRMT5 levels are continually kept under control and degraded through ubiquitin-dependent proteasomal degradation machinery. However, with Pt treatment, which causes higher overall cellular oxidative stress, KEAP1 is inhibited, and this inhibition leads to increased PRMT5 levels, which are needed for cells to survive the cellular stress induced by chemotherapeutic agents. Interestingly, although KEAP1 inhibition increased NRF2 levels, NRF2-depleted cells did not show increased sensitivity to carboplatin, indicating that the increased chemoresistance was not driven by increased NRF2. However, this may have been partially due to the limitations of 2D cell culture conditions. For example, NRF2 has been shown to drive the expression of selenoproteins (55, 56), which help control oxidative stress levels (57). However, selenoproteins require selenium to function, and selenium is typically absent in standard cell culture media. Consequently, while NRF2 activation may not appear essential in vitro, it remains to be tested whether it plays an important role in Pt resistance in vivo.

Although PRMTs are known to affect gene expression, the direction of this effect has been less understood. PRMT5 is generally categorized as a transcriptional repressor (20), probably because of its first discovery as part of a transcriptional repressor complex on the cyclin E1 promoter (58). However, recent findings contradict this broad labeling and indicate that PRMT5 positively contributes to gene expression (44). Notably, PRMT5 chromatin binding intensity aligns well with overall chromatin accessibility and active histone modifications, suggesting that PRMT5 positively regulates gene expression, at least in Pt-resistant cell settings.

PRMTs, specifically PRMT5, are generally upregulated in several cancers, suggesting their oncogenic tumor-promoting function. Our genomic analysis indicated that among all the cancers, ovarian and testis cancers have the highest levels of *PRMT5* mRNA expression, suggesting that PRMT5 is a viable therapeutic target in these cancers. Critically, our additional and mechanistic studies further revealed PRMT5 to be a key combinatorial therapeutic target in Pt-resistant cells, in which PRMT5 protein levels are further stabilized due to stress-induced KEAP1 inhibition. Supporting this hypothesis, we observe markedly higher cytotoxicity and apoptotic synergy between carboplatin and PRMT5 inhibitor treatment in Pt-resistant cells compared with chemo-naive cells, which have substantially lower PRMT5 levels. Of note, this also suggests that targeting PMRT5 in tumors with high levels of PRMT5 will have a notable therapeutic index. Therefore, combinatorial PRMT5 targeting in tumors with high PRMT5 levels can be a viable therapeutic avenue, in addition to targeting in tumors with *MTAP-CDKN2A* codeletion (53, 59). *MTAP* deletion leads to the accumulation of 5′-*O*-methylthioadenosine (MTA), which inhibits the binding of S-adenosylmethionine (SAM) to PRMT5, resulting in reduced PRMT5 enzymatic activity. As a result, tumors with *CDKN2A-MTAP* codeletion are selectively more sensitive to PRMT5 inhibitors (53, 59, 60), some of which are currently being tested in clinical trials (NCT03573310, NCT02783300, NCT03854227, and NCT03614728).

Importantly, recent reports from us and other groups have documented the pivotal role of PRMT5 in DNA repair (14–16), highlighting that targeting PRMT5 in combination with other DNA-damaging agents, but not alone, may have better therapeutic value. Supporting these findings, we show here that combined PRMT5 inhibition with a Pt DNA-damaging agent created synergistic cell death through increased DNA damage accumulation. Although these findings shed some light on what PRMT5 is doing inside cells, we do not quite understand why targeting PRMT5 creates a synergistic accumulation of DNA damage. Given the fast kinetics of DNA damage accumulation, this is probably not due to the transcriptional role of PRMT5. In line with this, we did not see significant Pt sensitization when more than 10 of the top PRMT5 target genes were depleted. Therefore, future studies must reveal why PRMT5 depletion creates rapid DNA damage accumulation in cells.

## Methods

### Sex as a biological variable.

Our study exclusively examined female mice because the disease modeled is only relevant in females.

### Cell culturing.

The human OC cell lines OVCAR4, Kuramochi, and A2780 were cultured in RPMI-1640 (11875093, Thermo Fisher Scientific) supplemented with 10% FBS (35011CV, Corning). HEK 293 cells were cultured in DMEM (11965118, Thermo Fisher Scientific) containing 10% FBS. COV362 cells (human OC cell line) were cultured in DMEM supplemented with 10% FBS and 1% GlutaMAX (35050061, Thermo Fisher Scientific), while the ID8 mouse ovarian surface epithelial cell line was cultured in DMEM containing 5% FBS. FT-190, a human telomerase reverse transcriptase (hTERT)-immortalized human fallopian tube secretory epithelial cell line, cells were cultured in DMEM/F12 50:50 Mix [–] l-glutamine (15-090-CV, Corning) supplemented with 10% FBS and 2 mM l-glutamine (25030081, Thermo Fisher Scientific). All media were supplemented with 1% penicillin/streptomycin (15140122, Thermo Fisher Scientific). The cells were maintained in a humidified incubator at 37°C. FT-190, Kuramochi, and ID8 cells were provided by Daniela Matei (Northwestern University, Evanston, Illinois, USA). A2780, OV81, COV362, and OVCAR4 cells were provided by Charles Landen (University of Virginia, Charlottesville, Virginia, USA). To generate Pt-resistant cell lines, A2780, OV81, and COV362 cells were treated with IC_50_ doses of cisplatin at regular intervals (6). Meanwhile, ID8 and OVCAR4 cells were continuously exposed to carboplatin and cisplatin, respectively. All cell lines developed at least 3- to 4-fold resistance to Pt treatment.

### Western blotting.

Cells were lysed using RIPA buffer (BP-115, Boston BioProducts), and protein concentrations were determined using a bicinchoninic acid (BCA) assay (23225, Thermo Fisher Scientific). Proteins (1 μg/μL) were mixed with 4× sample buffer (BP-110R, Boston BioProducts) and boiled at 95°C for 10 minutes. For Western blot analysis, 20 μg protein was loaded onto a NuPAGE 4% to 12% Bis-Tris gradient gel (NP0335, Thermo Fisher Scientific) and run at 140 V for approximately 1 hour. Proteins were transferred onto a nitrocellulose membrane using the iBlot dry transfer system. Next, membranes were blocked with 5% milk dissolved in TBS-T (20 mM Tris, 150 mM NaCl, 0.1% Tween 20; pH 7.6) for 1 hour at room temperature (RT). After blocking, membranes were incubated overnight at 4°C with primary antibodies prepared in the blocking buffer. The following primary antibodies were used in this study: anti-PRMT5 (1:2,000, ab210437, Abcam), anti-SDMA (1:1,000, 13222, Cell Signaling Technology [CST]), anti-Actin (1:10,000, a2228, MilliporeSigma), anti-P53 (1:1,000, 2524, CST), anti-KEAP1 (1:1,000, ab227828, Abcam), anti-ubiquitin (1:1,000, 3936S, CST), anti-FLAG (1:1,000, F3165, MilliporeSigma), anti-γ-H2AX (1:1,000, 9718, CST), and anti-FER (1:500, 4268, CST). The next day, membranes were washed 3 times with TBS-T for 5 minutes each wash. Next, they were incubated for 1 hour at RT with either anti–mouse HRP conjugate (1:10,000, W402B, Promega) or anti–rabbit HRP conjugate (1:10,000, W401B, Promega). After the incubation, membranes were washed 3 times for 10 minutes each. Finally, membranes were covered with Western blot detection reagents (37074, Thermo Fisher Scientific) and visualized using the Invitrogen iBright imaging system (Thermo Fisher Scientific). To isolate cytoplasmic and nuclear fractions, cells were lysed using a Cell Fractionation Kit following the manufacturer’s instructions (9038, CST). Proteins from each fraction were then analyzed by Western blotting as described above.

### qPCR.

RNA extraction was performed using the Quick-RNA MiniPrep kit (R1054, Zymo Research). After isolation, 1 μg RNA was converted to cDNA using the High-Capacity RNA-to-cDNA Kit (4387406, Thermo Fisher Scientific). The cDNA was then diluted at a 1:5 ratio using nuclease-free water. qPCR was performed using Fast SYBR Green Master Mix (4385616, Thermo Fisher Scientific) with 15 ng cDNA per reaction, and each reaction was performed in triplicate. qPCR results were obtained using the QuantStudio 3 system, and Ct values were analyzed using the 2^–ΔΔCt^ method (61). *GAPDH* expression was used as an internal control for normalization.

### Immunofluorescence.

Cells (1.5 × 10^5^) were seeded onto coverslips in 6-well plates. The next day, cells were treated with the 1 μM EPZ015666 (CS7748, Selleckchem) and 10 μM carboplatin (S1215, Selleckchem) for 3 days. After treatment, cells were washed with PBS and fixed using 4% paraformaldehyde in PBS for 10 minutes at RT. Following fixation, cells were washed 3 times with ice-cold PBS and incubated with 0.25% Triton X-100 in PBS for permeabilization. Next, cells were washed 3 times for 5 minutes each with PBS and blocked using 1% BSA and 22.52 μg/mL glycine in PBS plus 0.1% Tween 20 (PBS-T) for 1 hour at RT. After blocking, cells were incubated with anti-γ-H2AX (1:500, 9718, CST) antibody in 1% BSA in PBS-T overnight at 4°C in a humidified chamber. The next day, cells were washed 3 times for 5 minutes each with PBS-T and then incubated with anti–rabbit Alexa Fluor 594 (1:500, A-11012, Invitrogen, Thermo Fisher Scientific) in 1% BSA in PBS-T for 1 hour at RT. Subsequently, cells were washed 3 times for 5 minutes each with PBS-T. Coverslips were then mounted onto microscopy slides using mounting medium with DAPI (S36939, Thermo Fisher Scientific). Finally, slides were visualized using the EVOS cell imaging system (Thermo Fisher Scientific), and the images were analyzed using ImageJ software (NIH).

### Comet assay.

Microscopy slides were coated with 1.5% normal melting agarose to create a base layer. Cells were then counted and resuspended at a concentration of 1 × 10^5^ cells/mL. The cells were combined with low-melting agarose provided by the OxiSelect Comet Assay kit (STA-351, Cell Biolabs) at a 1:10 ratio, and 75 μL of this mixture was immediately transferred to the precoated slides and covered with a coverslip. After incubation at 4°C in the dark, the coverslips were carefully removed, and the slides were transferred to a container containing prechilled lysis buffer (provided by the kit). Following a 1-hour incubation with the lysis buffer at 4°C in the dark, the lysis buffer was replaced with prechilled alkaline solution (300 mM NaOH, 1 mM EDTA) and incubated for 30 minutes at 4°C in the dark. The slides were then transferred to an electrophoresis chamber filled with cold alkaline electrophoresis solution (prepared in-house) (300 mM NaOH, 1 mM EDTA, pH 13). Electrophoresis was performed at 17 V (1 volt/cm electrode distance) for 30 minutes at 4°C. After electrophoresis, the slides were washed 3 times for 2 minutes each with prechilled, distilled water. Subsequently, the slides were incubated once with cold 70% ethanol for 5 minutes and then air-dried at RT. Finally, the slides were stained with diluted Vista Green Dye (1:10,000 in Tris-EDTA buffer; STA-351, Cell Biolabs) for 15 minutes and visualized using the EVOS cell imaging system. Comet tails were measured using OpenComet software.

### Cell-cycle analysis.

Cells (1.5 × 10^5^ cells/well) were seeded in 6-well plates. The following day, cells were either mock treated or treated with 250 μM or 1,000 μM EPZ015666 for 72 hours. After the treatment, cells were harvested via trypsinization and fixed using 90% methanol with gentle vortexing. The fixed cells were incubated at 4°C for 30 minutes, followed by 2 washes with PBS. The cells were then stained with DAPI (1 mg/mL) for 30 minutes at RT. Finally, a minimum of 50,000 events were analyzed using a BD FACSMelody Cell Sorter, and the data were analyzed with FlowJo software.

### Lentivirus production and transduction.

To generate virus, 4 × 10^6^ HEK293 cells were seeded in a 10 cm dish. The next day, transgene (4 μg), psPAX2 (2 μg), and pmD2.G (1 μg) plasmids were transferred to an Eppendorf tube containing Opti-MEM (31985070, Thermo Fisher Scientific). Then, 21 μL of 1 mg/mL polyethylenimine (PEI) (24765, Polysciences) was added to the mixture (600 μL final volume), which was incubated for 15 minutes at RT. The mixture was then added dropwise to the cells. The following day, the media were replenished with fresh media, and the cells were incubated overnight. The next day, the culture media were collected and filtered using 0.45 μm filters (431225, Corning) and aliquoted for storage at –80°C.

For transduction, 1.25 × 10^5^ cells were seeded in a 6-well plate. The next day, the virus was thawed, and polybrene (TR-1003-G, MilliporeSigma) was added at a final concentration of 10 μg/mL. The cell media were replaced with viral media, and the cells were incubated overnight. The following day, the media were replenished with fresh media, and the cells were incubated for 48 hours. Finally, selection was performed using the appropriate selection marker until all uninfected cells were dead.

### CRISPR/Cas9-mediated gene manipulation.

For gene KO, at least 2 different sgRNAs were designed using CRISPOR software, selecting the lenti-guide-puro protocol. Each oligonucleotide pair (10 μM final concentration) was mixed in annealing buffer (10 mM Tris, pH 8, 50 mM NaCl, 1 mM EDTA) in a total volume of 50 μL and incubated at 95°C for 5 minutes. The mixture was then allowed to slowly cool to RT. The annealed oligonucleotides were diluted 1:200 with sterile water. A ligation reaction was performed using 50 ng BsmBI-v2 (R0739S, New England Biolabs [NEB]) digested backbone, 1 μL of the diluted oligonucleotides, and T4 ligase (M0202S, NEB), with incubation overnight at 16°C. The next day, 2.5 μL of the ligation reaction was transformed into stable, competent *E*. *coli* (C3040H, NEB) and incubated overnight in the presence of ampicillin selection. Several colonies were selected and grown overnight. The following day, plasmid DNA was isolated using the QIAprep Spin Miniprep Kit (27106, Qiagen) and sent for Sanger sequencing to validate successful insertion. After subcloning the sgRNAs, lentivirus was generated using HEK 293 cells as explained above. Cell lines were then infected with these viruses and selected with 1 μg/mL puromycin (ant-pr-1, InvivoGen) until all uninfected cells were killed. After selection, cells were maintained for an additional 3 days, and then proteins were extracted using RIPA buffer. Finally, Western blotting was performed to determine the level of protein downregulation.

To achieve epigenetic downregulation of PRMT5 expression, a parental cell line expressing the dCas9-KRAB protein was first generated by infecting cells with a virus encoding the dCas9-KRAB protein. Simultaneously, sgRNAs targeting PRMT5 promoter regions were designed and subcloned using the same strategy described above. After delivering the sgRNAs, qPCR and Western blot analyses were performed to confirm PRMT5 downregulation.

### Cell viability assays and synergy calculations.

To determine cell viability, 1 × 10^3^ cells/well were seeded in 96-well plates (3610, Corning). The next day, the cells were treated with different doses of EPZ015666 and carboplatin and incubated for 6 days. After incubation, the media were aspirated, and 44 μL CellTiter-Glo 2.0 Reagent (G9242, Promega) diluted 1:10 with colorless media (11058021, Thermo Fisher Scientific) was added to each well (40 μL media, 4 μL reagent). The luminescence signal was then recorded using a plate reader, and the results were analyzed with GraphPad Prism software. To assess the synergistic response, the SynergyFinder package in RStudio was used (62), along with the Bliss method (48).

### Apoptosis assay.

Cells were seeded in a 96-well plate at a density of 4 × 10^3^ cells/well. The following day, treatments were administered using a drug of interest mixed with a 1:1,000 dilution of caspase 3/7 dye (10403, Biotum) and a 1:2,000 dilution of SiRDNA nuclei stain (CY-SC007, Spirochrome). Subsequently, cells were monitored with the IncuCyte (Sartorius) live imaging system, using phase, green, and red channels. The apoptosis rate was determined using the green integrated intensity/red object count, and the results were plotted in GraphPad Prism.

### Analysis of public datasets.

PRMT5 correlation values in HGSOC were obtained from the cBioPortal. The data were ranked according to correlation values and plotted using ggplot2. Positively correlated genes were identified on the basis of a Spearman correlation value of greater than 0.5 and a FDR of less than 0.001. These identified genes were then analyzed using DAVID GO term analysis, and the resulting terms were plotted in RStudio.

TCGA dataset was used to illustrate PRMT5 expression across various tumors, with expression values plotted using the ggplot2 package in RStudio. Comparison of PRMT5 expression levels between the fallopian tube, ovary, and ovarian serous cystadenocarcinoma (OSC) was conducted using the UCSC Xena Browser. Additionally, progression-free survival plots were generated with the Kaplan-Meier plotter online tool (https://kmplot.com/analysis/), using the following criteria: split patients by – median, histology - serous, stage - all, grade - 3, *TP53* mutation - mutated.

### TMA staining.

TMAs were stained using anti-PRMT5 antibody (ab210437, Abcam) following the manufacturer’s instructions. The TMA was analyzed with QuPath software (38). Initially, TMA dearray was used to correctly assign annotations with the metadata. Subsequently, PRMT5 staining intensities were detected in individual cells. Following this, QuPath’s machine learning algorithm was utilized to distinguish between stroma and tumor cells. Finally, the staining intensities were converted to H-score values and plotted with ggplot2 in RStudio.

### CRISPR screening using the druggable genome library.

Cells were initially transduced with a Cas9-expressing construct. Following the generation of Cas9-expressing cells, the druggable genome library was transduced at approximately 0.3 MOI. Approximately 15 × 10^6^ cells were transduced to achieve 500× coverage. The next day, the media were replenished, and puromycin (1 μg/mL) was added 48 hours later for selection. After selection, cells were split into 4 replicates and passaged every 4–5 days until confluent. After 2 weeks, they were trypsinized and resuspended in 200 μL PBS. Subsequently, they were fixed using 200 μL of a 1% formaldehyde solution while vortexing and then incubated for 10 minutes at 37°C. For permeabilization, ice-cold absolute methanol was added dropwise, and the cells were incubated for 30 minutes at 4°C, after which they were washed twice with PBS and resuspended in 400 μL PBS containing 2% BSA (PBS-BSA). Then, 1 μL anti–PE-PRMT5 (ab210437, Abcam) antibody was added, and the cells were incubated on ice for 30 minutes. After incubation, the cells were washed twice with PBS-BSA and resuspended in 4 mL PBS-BSA solution. The cells were then sorted to obtain populations with high (top 20%) and low (bottom 20%) PRMT5 levels using the BD FACSMelody Cell Sorter. The sorted cells were resuspended in lysis buffer (10 mM Tris-HCl, 150 mM NaCl, 10 mM EDTA, 0.1% SDS) supplemented with proteinase K (500 μg/mL, EO0491, Thermo Fisher Scientific) and RNase A (100 μg/mL, EN0531, Thermo Fisher Scientific) and incubated for 24 hours at 55°C. The following day, DNA was extracted using the standard phenol-chloroform extraction protocol. Libraries were prepared using the NEBNext High-Fidelity 2× PCR Master Mix (M0541L, NEB) PCR protocol with custom-barcoded oligonucleotides. In total, 11 PCR reactions per replicate were performed using 1 μg DNA per reaction. DNA was amplified for 25 cycles and loaded onto an agarose gel. Bands corresponding to the libraries were excised and purified using the QIAquick gel extraction kit (28704, Qiagen). Finally, the libraries were pooled and sequenced on the Illumina NextSeq 500 platform to obtain single-end reads. To analyze the reads, the CB2 package was utilized according to the package instructions in RStudio (40). Briefly, reads were mapped to a FASTA file containing sgRNA library sequences. The aligned reads were then used to calculate the average read count per sgRNA. After ensuring that each sgRNA had enough coverage (<500 reads per sgRNA), the data were normalized using counts per million (CPM) normalization. Subsequently, CPM-normalized reads were statistically compared using the measure_sgrna_stats function in CB2. Finally, sgRNA statistics were converted to gene-level enrichments using the “measure_gene_stats function.” Genes were assigned significance for PRMT5^hi^ on the basis of a log_2_ fold change (FC) of greater than 0.25 and a *P* value of less than 0.01, and for PRMT5^lo^, a log_2_ FC of less than –0.25 and a *P* value of less than 0.001. Plots were generated using ggplot2.

### Co-IP assay.

HEK 293 cells (5 × 10^6^) were seeded in 10 cm dishes and transfected with 5 μg FLAG-KEAP1 plasmid. MG132 (1 μM, M7449, MilliporeSigma) was added after 24 hours. At 48 hours, cells were washed once with PBS and collected by scraping. Following centrifugation, the cell pellet was lysed in 1 mL co-IP buffer (50 mM Tris, pH 8.0, 0.5% NP-40, 200 mM NaCl, 0.1 mM EDTA, 10% glycerol, and protease inhibitors). The samples were then incubated on ice for 30 minutes and centrifuged at 15,000*g* for 20 minutes at 4°C. Subsequently, 75 μL lysate was transferred into a new Eppendorf tube and mixed with 25 μL sample buffer to obtain whole-cell extract. To the remaining lysate, 2 μg Flag antibody (F3165, MilliporeSigma) was added and incubated overnight at 4°C with rotation. The next day, 30 μL protein A/G magnetic beads (88802, Thermo Fisher Scientific) per sample were equilibrated with co-IP buffer (by washing 3 times with 1 mL buffer) and added to the samples. After a 2-hour incubation at 4°C, the beads were immobilized and washed 3 times with 1 mL co-IP buffer and once with PBS. Finally, the beads were resuspended in 60 μL 2× sample buffer and boiled at 95°C for 10 minutes, and the samples were analyzed by SDS-PAGE.

### IP analysis.

HEK 293 cells were seeded in 10 cm dishes and transfected the next day with PRMT5 (5 μg), KEAP1 (5 μg), and ubiquitin plasmids (5 μg). Vector plasmid was also used to equalize transfection amounts. The following day, cells were treated overnight with either 1 μM MG132 or DMSO. Next, cells were scraped in ice-cold PBS and subsequently lysed using 2% SDS and 50 mM Tris, pH 8. Afterwards, they were briefly sonicated and then diluted 1:10 using IP buffer (50 mM Tris, pH 8, 200 mM NaCl, 0.1 mM EDTA, 0.5% NP-40, 10% glycerol, and protease inhibitors). Lysates were incubated with 5 μg anti-PRMT5 antibody (07-405, MilliporeSigma) at 4°C overnight with rotation. The next day, protein A/G magnetic beads were equilibrated in IP buffer and added to the lysates (30 μL/sample). After incubating the lysate-bead complex at 4°C for 2 hours, the beads were washed 4 times using IP buffer and once with PBS. Finally, the beads were resuspended in 2× sample buffer and boiled at 95°C for 10 minutes. The samples were subjected to Western blotting as explained above.

### In vivo xenograft experiment.

For the in vivo experiments, we used a PDX model developed by Dong et al. (63). The primary clinical and pathological characteristics of this model include high-grade serous carcinoma (HGSC), stage T3C, with pathologic lymph node status cannot be assessed (pNX) following total abdominal hysterectomy and bilateral salpingo-oophorectomy. The primary tumor measured 7 cm in diameter, and it harbored a p53 mutation. The patient’s age at diagnosis was not recorded. More detailed histological and molecular analyses of the PDX model are provided in the article. To create a carboplatin-resistant PDX model for this study, PDX tumor–engrafted mice were treated with carboplatin for approximately 4 weeks, after which tumor regrowth was allowed. The tumors were then passaged into new recipient mice and treated with carboplatin again. This cycle of carboplatin treatment was repeated at least 4 times to ensure the development of resistance. Six- to 7-week-old female NSG mice were obtained from The Jackson Laboratory (strain no. 005557). All mice were housed in a pathogen-free animal barrier facility. PDX tumors were s.c. engrafted into NSG mice. When the tumors reached a size of 100–250 mm^3^, the animals were randomly assigned to 4 groups. Carboplatin (HY-100235, MedChemExpress) was administered at 10 mg/kg once a week, while GSK591 (S1215, Selleckchem) was administered at 50 mg/kg 5 days a week. Both treatments were given via i.p. injection. Tumor growth was measured every 2–3 days using calipers. Animals were sacrificed when tumors reached approximately 1,500 mm^3^ in size.

### ChIP with high-throughput sequencing.

Cells (5 × 10^6^) were seeded onto 15 cm dishes and grown until approximately 90% confluence. Proteins were crosslinked to DNA by adding formaldehyde (1% final concentration), followed by a 15-minute incubation at RT. The crosslinking reaction was then stopped by adding glycine (125 mM final concentration) to each sample and incubating for 5 minutes at RT. Media were aspirated, and cells were washed twice using 5 mL ice-cold PBS containing protease inhibitors. Cells were then scraped into 5 mL PBS and centrifuged at 4°C for 10 minutes at 1650*g*. Next, cells were resuspended in 1 mL SDS lysis buffer (1% SDS, 10 mM EDTA, 50 mM Tris-HCl, pH 8.1) and incubated on ice for 10 minutes. Following incubation, DNA was sonicated to obtain 100–400 bp fragments using a Diagenode Bioruptor sonicator (7 cycles, 30 seconds on, 30 seconds off). Sonicated samples were centrifuged at 24°C for 10 minutes at maximum speed. Samples were then diluted 1:5 using ChIP dilution buffer (0.01% SDS, 1.1% Triton X-100, 1.2 mM EDTA, 16.7 mM Tris-HCl, pH 8.1, 167 mM NaCl, protease inhibitors). Whole-cell extract (250 μL) was transferred to an Eppendorf tube and stored at 4°C for DNA extraction. Anti-PRMT5 (5 μg) (07-405, MilliporeSigma) and anti-H3K27ac (2 μg) (ab4729, Abcam) antibodies were added to the samples, which were incubated at 4°C overnight on a rotator. The next day, 30 μL protein A or G Dynabeads (10001D-10003D, Thermo Fisher Scientific) per sample were equilibrated with ChIP dilution buffer and added to the samples. After a 2-hour incubation at 4°C, Dynabeads were immobilized using a magnetic stand and washed twice with 1 mL Low Salt Immune Complex Wash Buffer (0.1% SDS, 1% Triton X-100, 2 mM EDTA, 20 mM Tris-HCl, pH 8.1, 150 mM NaCl). Beads were then washed twice with 1 mL LiCl Immune Complex Wash Buffer (0.25 M LiCl, 1% NP-40, 1% deoxycholate, 1 mM EDTA, 10 mM Tris-HCl, pH 8.1). Next, beads were washed twice with 1 mL TE buffer (10 mM Tris-HCl, 1 mM EDTA, pH 8.0). Beads were then resuspended in 125 μL elution buffer freshly supplemented with DTT (1× TE, pH 8.0, 1% SDS, 150 mM NaCl, 5 mM DTT) and incubated at 65°C for 10 minutes. Beads were immobilized, and eluted samples were transferred to a new tube. The elution step was repeated to obtain a total of 250 μL eluted samples. Ten microliters of 5 M NaCl, 25 μL of 10% SDS, and 1.25 μL of 1 M DTT were added to the whole-cell extract samples. Samples were incubated at 65°C overnight for reverse cross-linking. The next day, proteinase K (500 μg/mL, EO0491, Thermo Fisher Scientific) was added, and samples were incubated at 55°C for 2 hours. Finally, DNA was extracted from the samples using phenol-chloroform extraction. To make libraries, the NEBNext Ultra II DNA Library Prep Kit for Illumina (E7645S, NEB) was used following the manufacturer’s instructions. Libraries were sequenced using the Illumina HiSeq 4000 to obtain 50 bp single-end reads.

### ATAC-Seq.

Cells were trypsinized and counted to obtain 50,000 cells/sample. Cells were washed once with ice-cold PBS and centrifuged at 500*g* for 5 minutes at 4°C. Nuclei were extracted by resuspending the cells in 50 μL ATAC-Seq lysis buffer (10 mM Tris-HCl, 10 mM NaCl, 3 mM MgCl_2_, 0.1% Tween 20, 0.1% NP-40, 0.01% digitonin) and incubating on ice for 3 minutes. Next, 1 mL wash buffer (10 mM Tris-HCl, 10 mM NaCl, 3 mM MgCl_2_, 0.1% Tween 20) was added, and the tubes were inverted 3 times. Samples were centrifuged at 500*g* for 10 minutes at 4°C. The supernatant was discarded, and nuclei were dissolved in 50 μL transposition mix (25 μL 2× TDE buffer, 2.5 μL TDEI Tn5 enzyme, 16.5 μL PBS, 0.5 μL 1% Digitonin, 0.5 μL 10% Tween 20, 5 μL nuclease-free water), with pipetting done 6 times. Transposition was carried out for 30 minutes at 37°C. Next, transposed DNA was eluted using the Qiagen MinElute kit (28004, Qiagen) according to the manufacturer’s instructions. To prepare libraries, 13 cycles of PCR were performed using custom oligonucleotides as described before (64). Libraries were purified by double-sided bead purification using AMPure beads (A63881, Beckman Coulter). Libraries were pooled and sequenced on an Illumina NextSeq 500 to obtain 50 million paired-end reads per sample.

### RNA-Seq.

Cells (2 × 10^5^) were seeded into 6-well plates in triplicate. The next day, the cells were treated with either DMSO or 5 μM EPZ015666 for 5 days. RNA was extracted using the Quick-RNA Miniprep Kit (R1054, Zymo Research). For library preparation, 1 μg input RNA was used following the NEBNext Ultra II Directional RNA Library Prep Kit for Illumina (E7760S, NEB) protocol. The prepared libraries were pooled and sequenced on an Illumina HiSeq 4000.

### Data analysis.

All sequencing data were first quality checked using FastQC. ATAC-Seq reads were aligned to the T2T reference genome using Bowtie2 software (settings: --local --very-sensitive) (65). Reads aligned to the mitochondrial genome were then removed using Samtools (66). PCR duplicates and blacklisted regions were removed using Picard and Bedtools, respectively. Peak calling was performed using MACS2 (settings: -f BAMPE --call-summits --keep-dup all) (67). BigWig files were generated using deeptools (settings: --binSize 1 --normalizeUsing RPGC). Genomic tracks were visualized on the UCSC Genome Browser. Heatmaps were generated using deeptools bamCoverage (settings: -a 2000 -b 2000 --skipZeros --missingDataAsZero --referencePoint center) (68). For ChIP-Seq reads, a similar pipeline was used, as with ATAC-Seq, except mitochondrial reads were kept. To identify peaks, tag directories were created using the HOMER makeTagDirectory function (69). PRMT5 binding regions were then identified with the findPeaks function, applying a FDR threshold of less than 0.001. Peaks were annotated using HOMER.

For RNA-Seq analysis, reads were aligned using STAR (settings: --quantMode GeneCounts --outSAMtype BAM SortedByCoordinate --outSAMunmapped Within --outSAMattributes Standard) (70). Subsequently, a count matrix file was generated from the STAR output. To determine differentially expressed genes, the DESeq2 package was used with default settings (71). Resistant cells were used as a reference sample, and genes with a FDR of less than 0.05 were assigned as differentially expressed. For visualization, BigWig files were generated using deeptools bamCoverage (settings: --binSize 1 --normalizeUsing RPKM) (68) and tracks were displayed on the UCSC Genome Browser.

### Statistics.

Statistical analyses were performed using GraphPad Prism 10 (GraphPad Software). Normality was assessed through the Shapiro-Wilk test. Differences between 2 groups were evaluated using 2-tailed Student’s *t* test or the Wilcoxon rank-sum test where appropriate. For comparisons of 3 or more groups, 1-way ANOVA with Dunnett’s multiple-comparison test was used. The tumor growth data were analyzed by 2-way ANOVA. Statistical significance was defined as a *P* value of less than 0.05. Data represent the mean ± SEM or SD, as indicated in the figure legends.

### Study approval.

All animal experiments and procedures complied with ethics regulations of the IACUC of Northwestern University under approved protocol no. IS000007992.

### Data availability.

All data used to generate the graphs are provided in the Supporting Data Values file. All genomic data generated in this manuscript have been deposited in the NCBI’s Gene Expression Omnibus (GEO) database (GEO GSE282674, GSE282675, GSE282676, and GSE282677).

## Author contributions

MA and HO conceptualized the study and wrote the manuscript. SO contributed to the TMA staining and analysis. HO performed the majority of the experiments and conducted the data analysis. FSP helped with flow cytometric analysis and CRISPR screening. NA and YK assisted with Western blot and qPCR experiments.

## Supplementary Material

Supplemental data

Unedited blot and gel images

Supporting data values

## Version 1 03/17/2025

Electronic publication
