# Supplementary material for: High PRMT5 levels, maintained by KEAP1 inhibition, drive chemoresistance in high-grade serous ovarian cancer
Source: J Clin Invest. 2025 Mar 17;135(6):e184283. doi: 10.1172/JCI184283 (PMC11910213; doi:10.1172/JCI184283)
Supplement: Supplemental data [file jci-135-184283-s139.pdf]

Supplementary Figures:

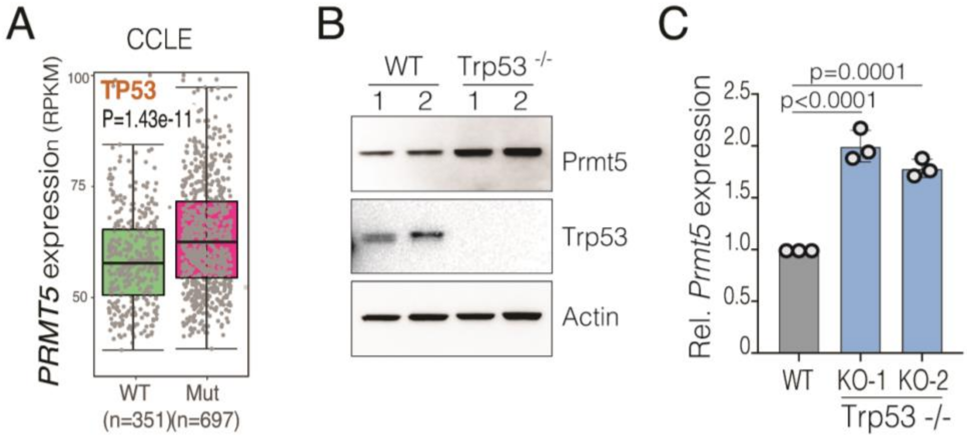

Supplementary Figure 1: (**A**) The box plot show *PRMT5* mRNA expression in TP53 wild-type (WT) and mutant cancer cell lines. Statistical significance was determined using the Wilcoxon Ranked Sum test. (**B**) Western blots display Prmt5 levels in Trp53-depleted ID8 cells, with actin used as a loading control. (**C**) The bar plot illustrates *Prmt5* mRNA expression in Trp53 WT versus Trp53 knockout (KO) ID8 cells. Data are presented as mean ± SD (n = 3). P-values were quantified by one-way ANOVA with Dunnett's multiple comparisons.

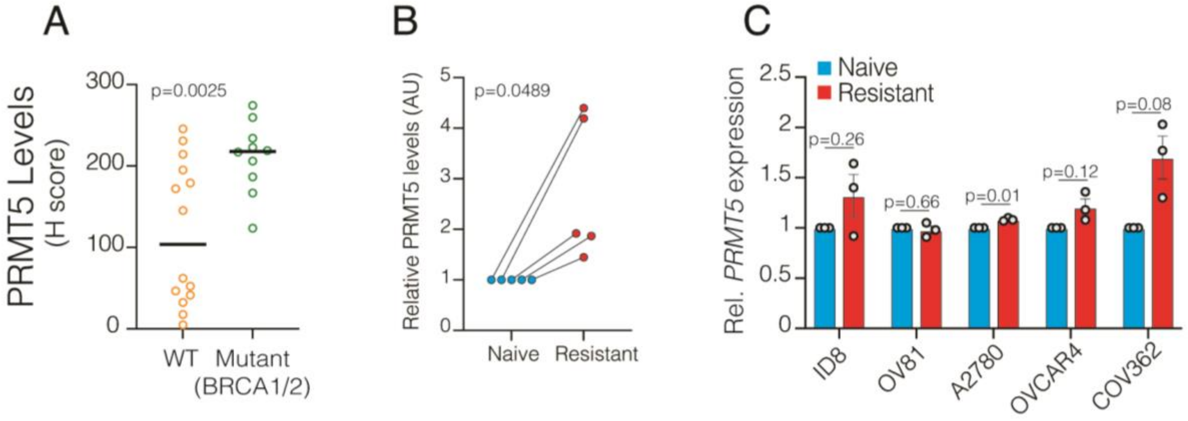

Supplementary Figure 2: (**A**) The dot plot shows PRMT5 staining levels in WT versus BRCA1/2 mutant primary tumors. P-values were determined using the two-tailed, unpaired Student's t-test. (**B**) The dot plot displays PRMT5 protein levels in Pt-naive and Pt-resistant isogenic cell lines. P-value was determined using the two-tailed, paired Student's t-test. (**C**) The bar plot shows relative PRMT5 mRNA expression between Pt-naive and Pt-resistant isogenic cell lines. Data are presented as mean ± SEM (n = 3). P-values were determined using the two-tailed, unpaired Student's t-test.

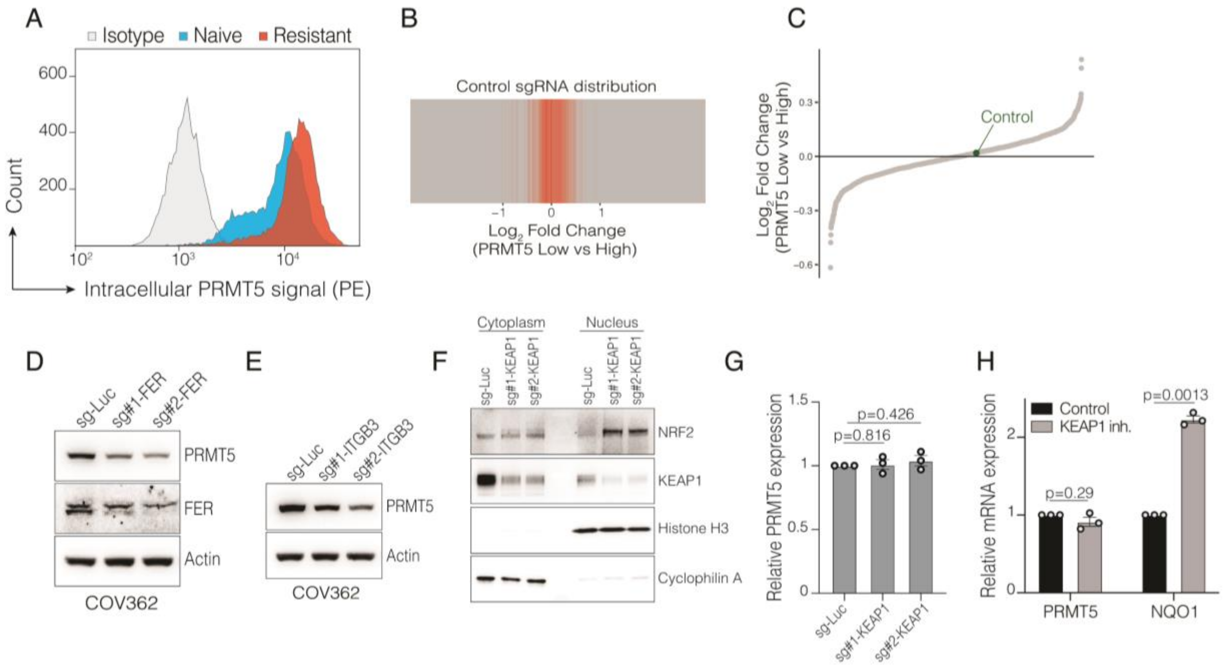

Supplementary Figure 3: (**A**) The density plot shows intracellular PRMT5 protein levels in naive and resistant cells, as determined by FACS. (**B**) The line plot illustrates the fold change of control sgRNAs included in the CRISPR knockout library. (**C**) The dot plot displays the enrichment of control sgRNAs. (**D-E**) Western blots display PRMT5 levels upon depletion of the *FER* (**D**) and *ITGB3* (**E**) genes. (**F**) Western blots show NRF2 levels in control (luciferase) and KEAP1 sgRNA-expressing cells. Histone H3 serves as a loading control for the nuclear fraction, while Cyclophilin A is used as a loading control for the cytoplasmic fraction. (**G**) The bar plot shows PRMT5 mRNA levels in control and KEAP1 sgRNA-expressing cells. Data are presented as mean ± SEM (n = 3). P-values were determined using the two-tailed, unpaired Student's t-test. (**H**) The bar plot shows *PRMT5* and *NQO1* mRNA levels upon KEAP1 inhibition. Cells were treated with 2 µM bardoxolone for 3 days. Data are presented as mean ± SEM (n = 3). P-values were determined using the two-tailed, unpaired Student's t-test.

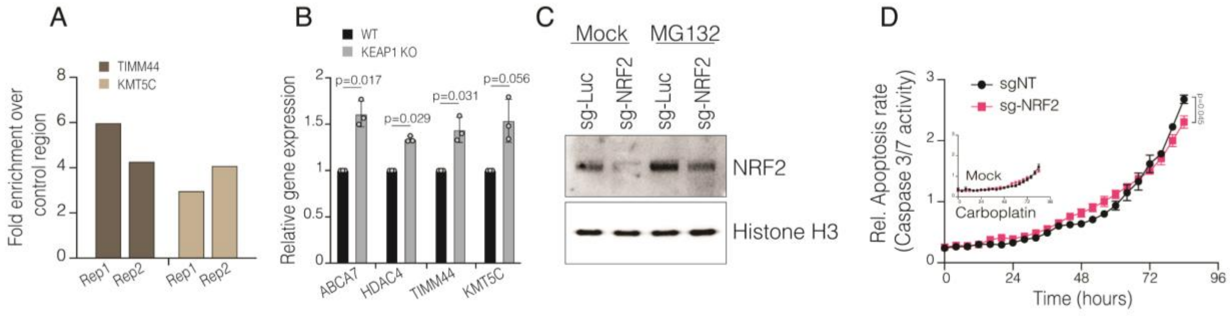

Supplementary Figure 4: (**A**) The bar plot illustrates PRMT5 enrichment at the promoter regions of the indicated genes, with the control region selected based on PRMT5 ChIP-seq data. (**B**) The bar plot shows mRNA expression levels of the indicated genes in WT and KEAP1 KO cells. Data are presented as mean ± SD (n = 3), and p-values were calculated using a two-tailed, unpaired Student's t-test. (**C**) Western blots show NRF2 protein levels in control and sg-NRF2-expressing cells. Overnight MG132 treatment (1 µM) applied to stabilize NRF2 protein levels. (**D**) The line plot displays relative apoptosis levels in control and sg-NRF2-expressing cells. Cells were treated with 20 µM carboplatin, and apoptosis was monitored over 84 hours using the IncuCyte live-cell imaging platform. Data are presented as mean ± SEM (n = 3), and p-values were determined using a two-tailed, unpaired Student's t-test.

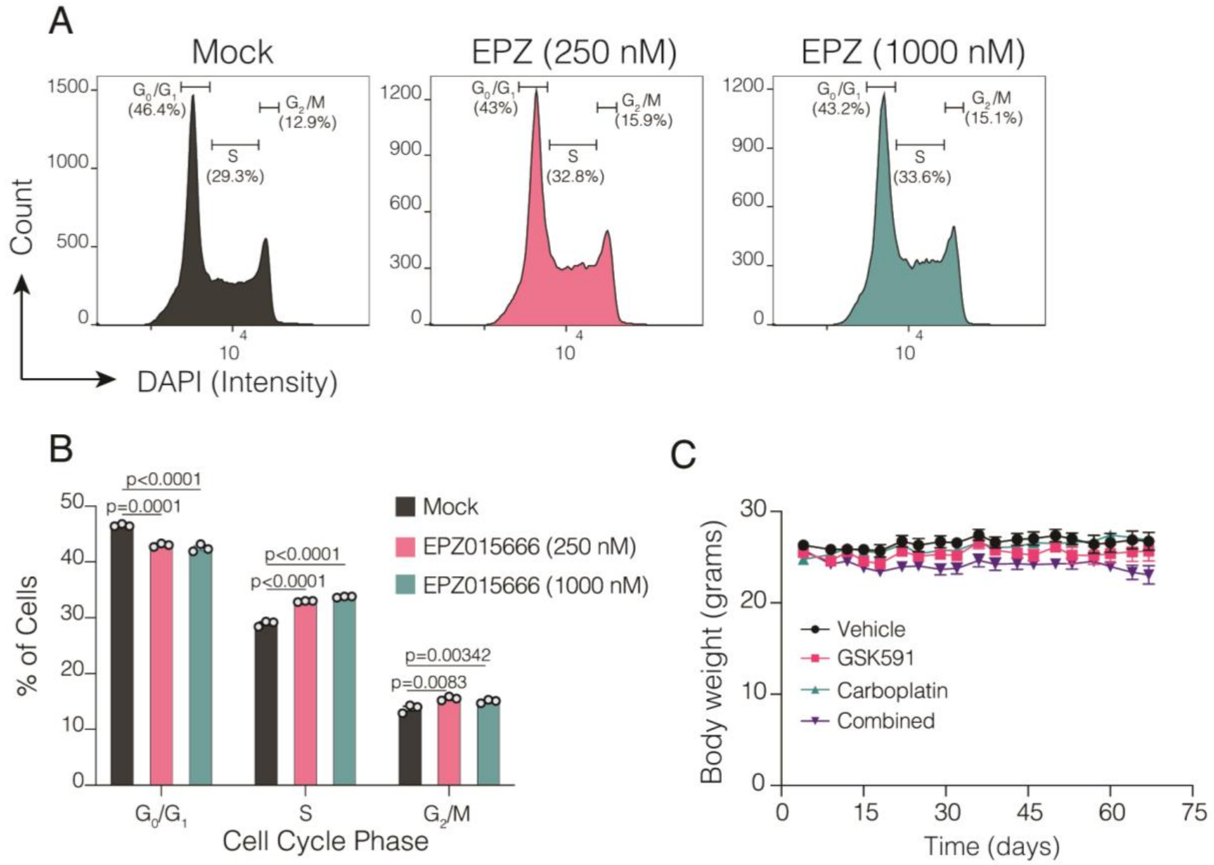

Supplementary Figure 5: (**A**) Density plots display DAPI intensity and corresponding cell counts after 72 hours of treatment with Mock or EPZ015666 at concentrations of 250 µM and 1000 µM. Cell cycle phases were determined using FlowJo software. (**B**) Bar plots display the distribution of cell cycle phases as determined in A. Data are presented as mean ± SEM (n = 3), and p-values were quantified by one-way ANOVA with Dunnett's multiple comparisons. (**C**) The line plot shows body weight measurements of mice over a ~60-day treatment period. Mice were administered vehicle, GSK591 (50 mg/kg), carboplatin (10 mg/kg), and their combination for approximately 60 days. Data are presented as mean ± SEM.
