# Supplementary material for: High PRMT5 levels, maintained by KEAP1 inhibition, drive chemoresistance in high-grade serous ovarian cancer
Source: J Clin Invest. 2025 Mar 17;135(6):e184283. doi: 10.1172/JCI184283 (PMC11910213; doi:10.1172/JCI184283)
Supplement: Unedited blot and gel images [file jci-135-184283-s140.pdf]

Fig. 2E

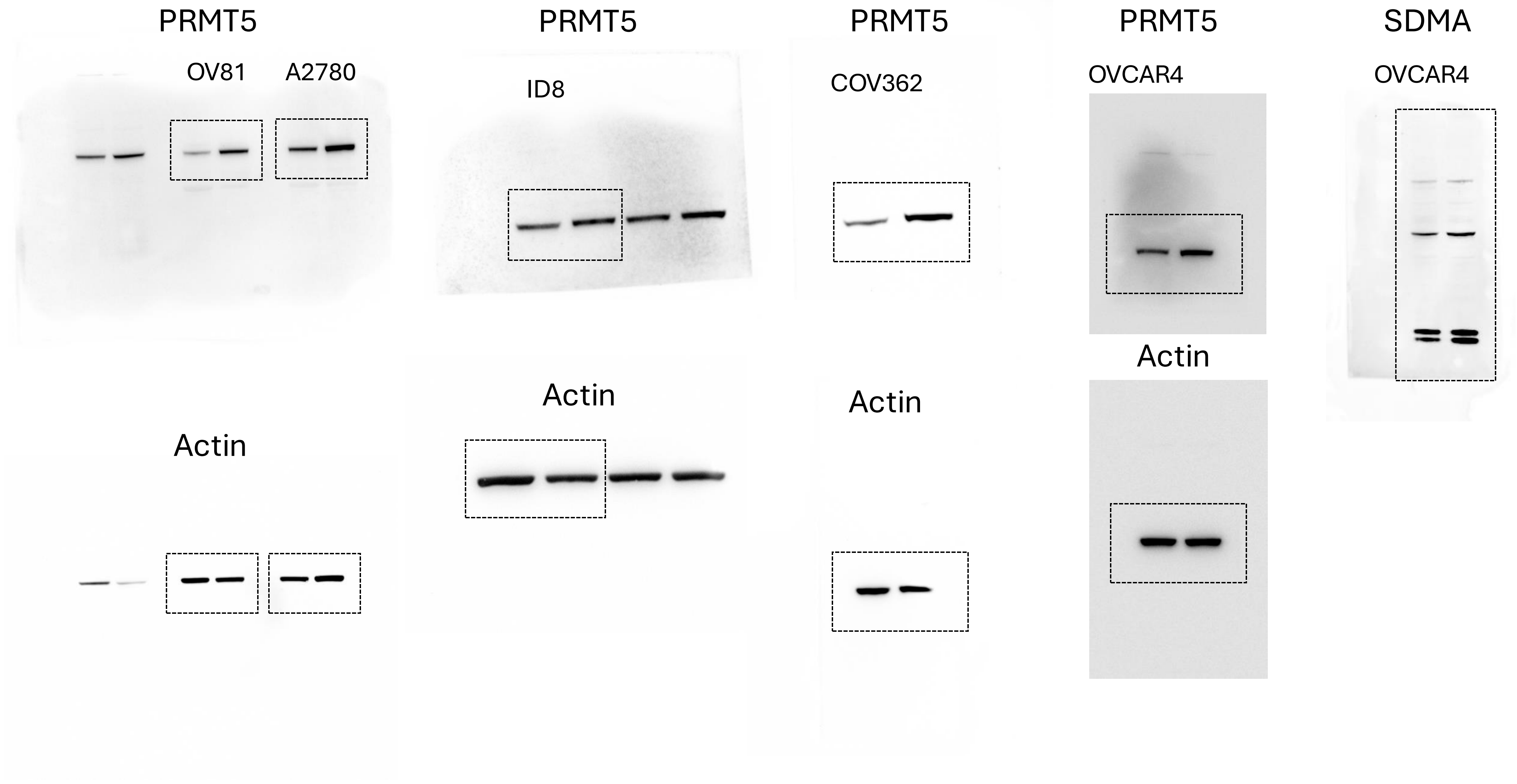
PRMT5
OV81
A2780
Actin
PRMT5
ID8
Actin
PRMT5
COV362
Actin
PRMT5
OVCAR4
Actin
SDMA
OVCAR4

Fig. 2E

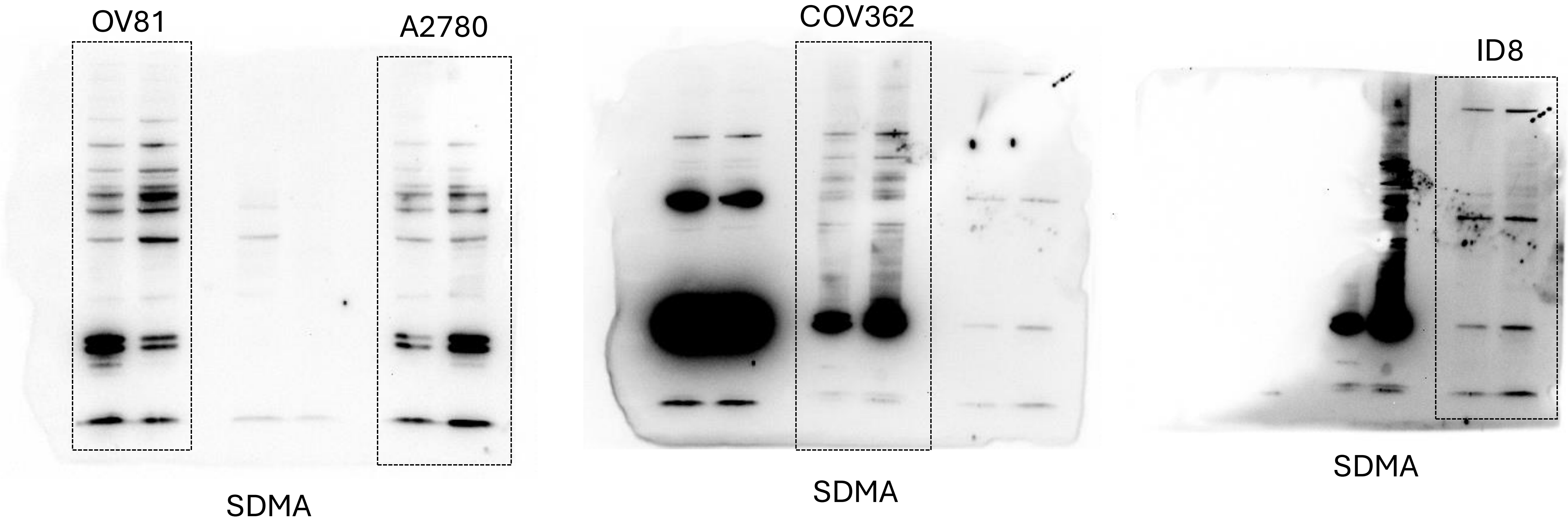

Fig. 2F

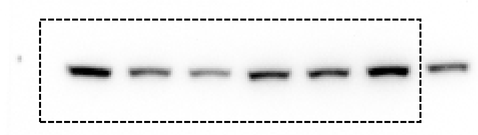

PRMT5

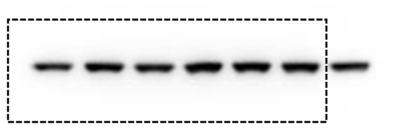

Actin

Fig. 2H

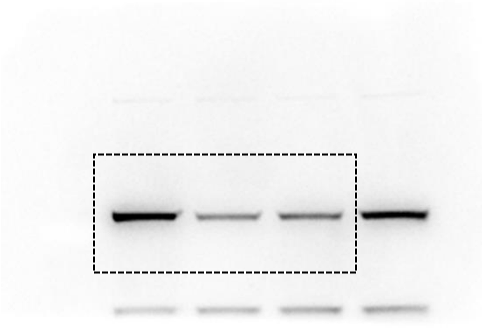

PRMT5

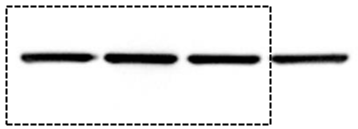

GAPDH

Fig. 3D

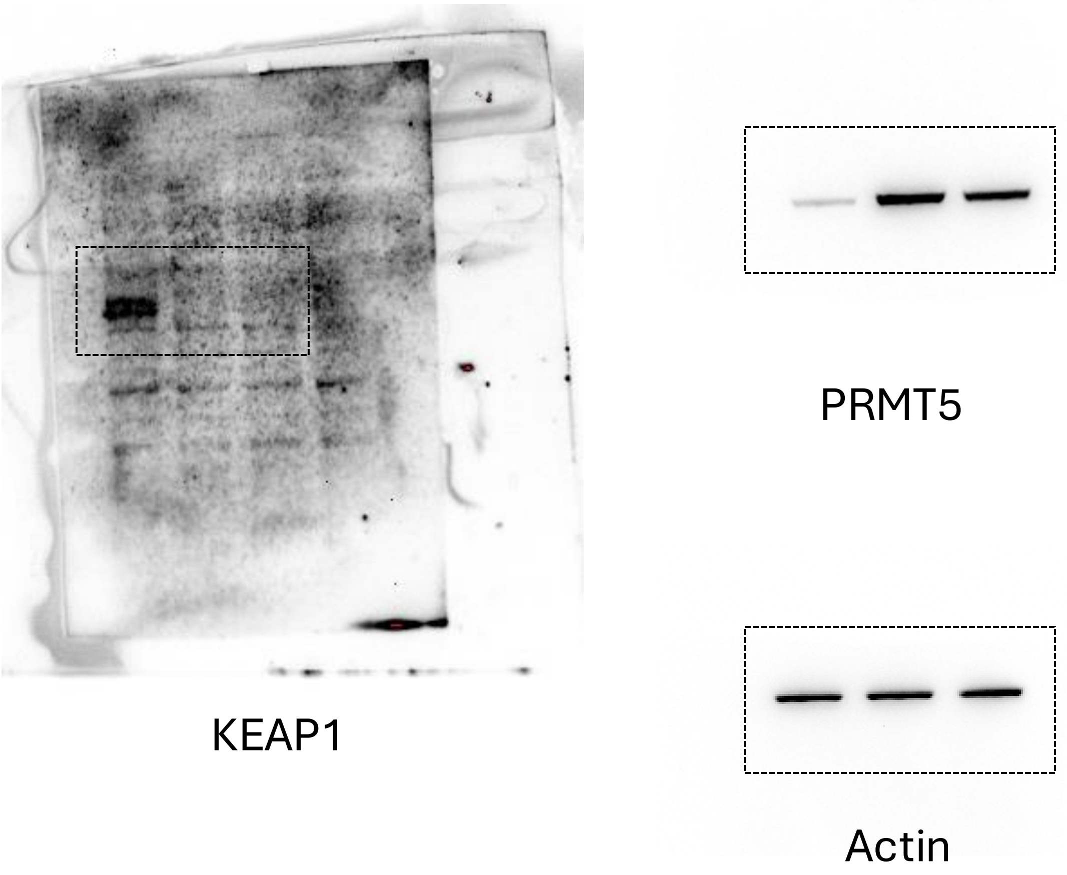

Fig. 3E

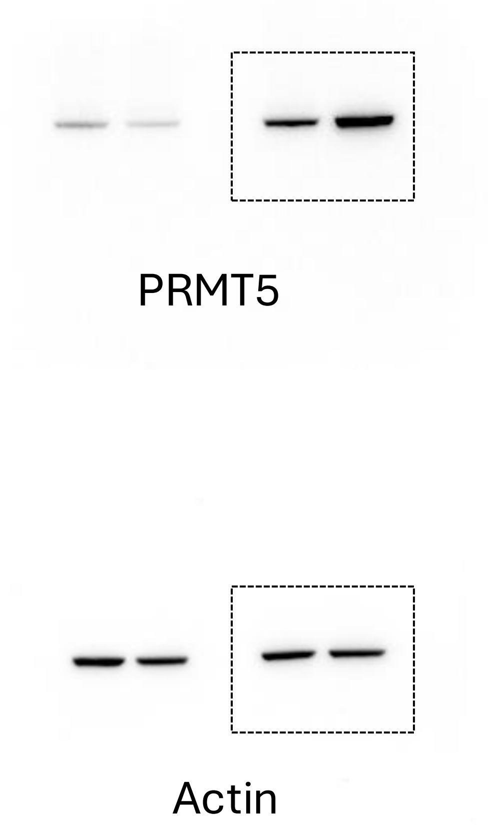

Fig. 3F

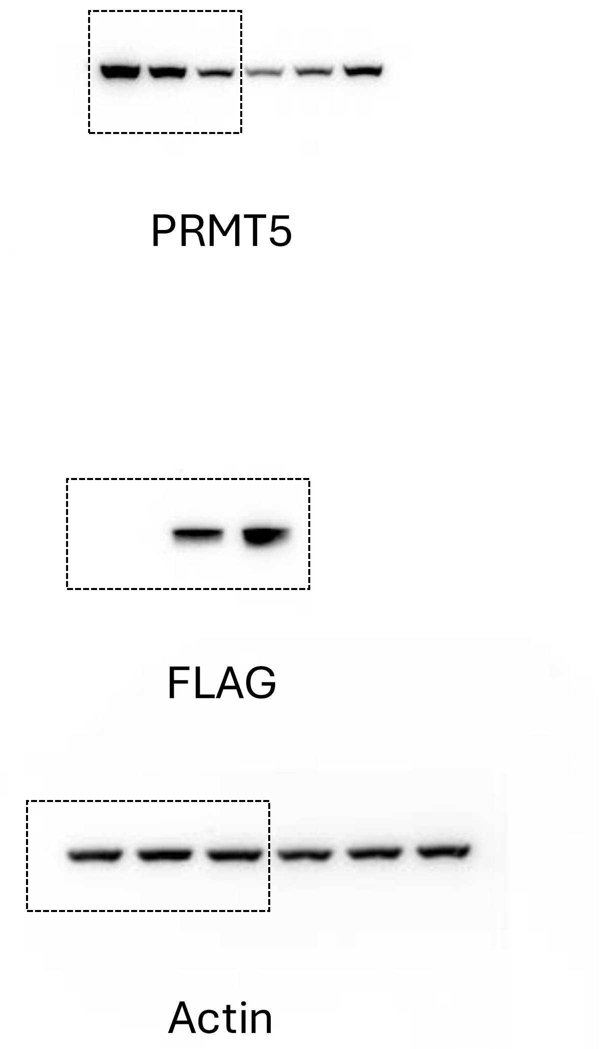

Fig. 3G

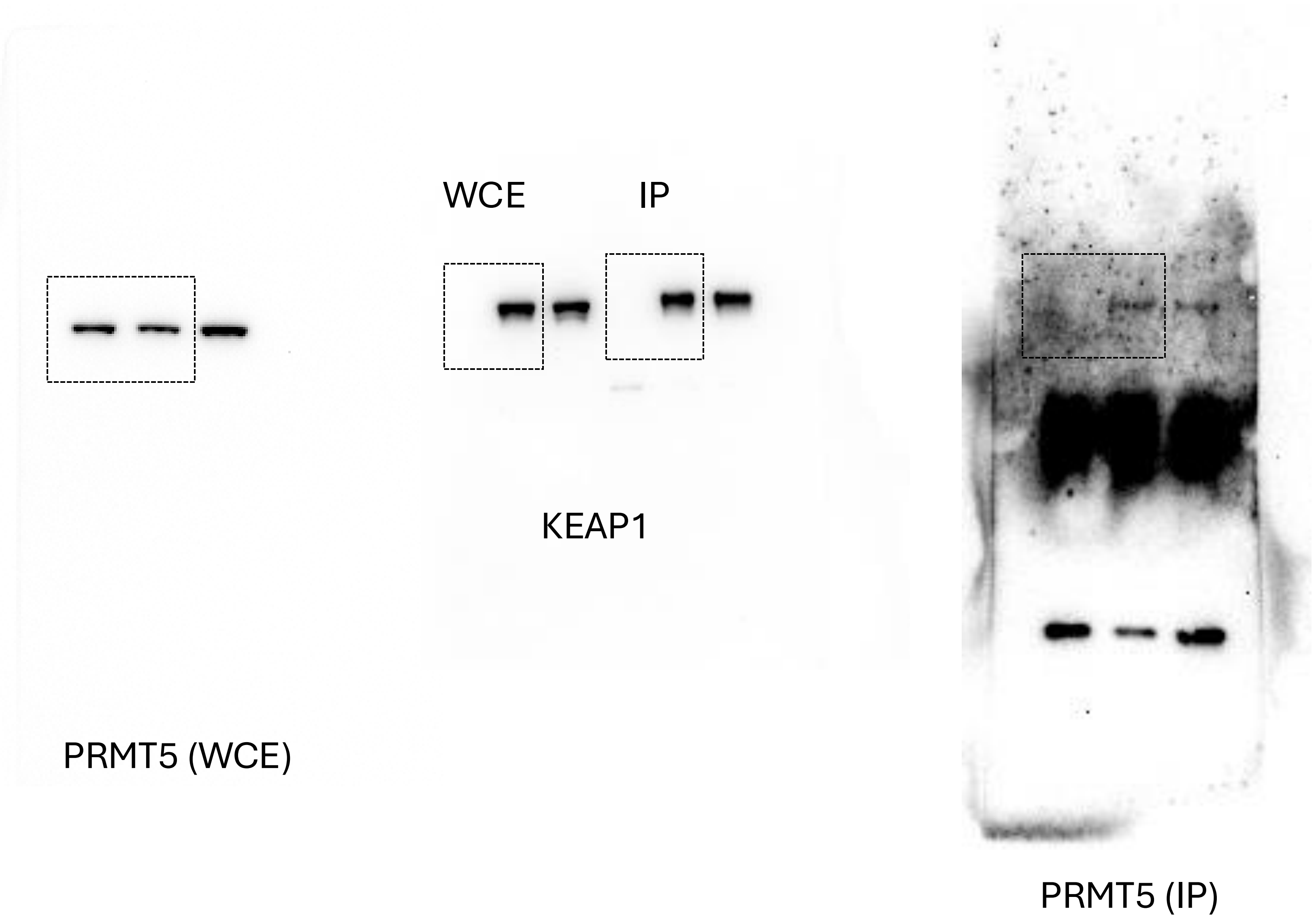

Fig. 3H

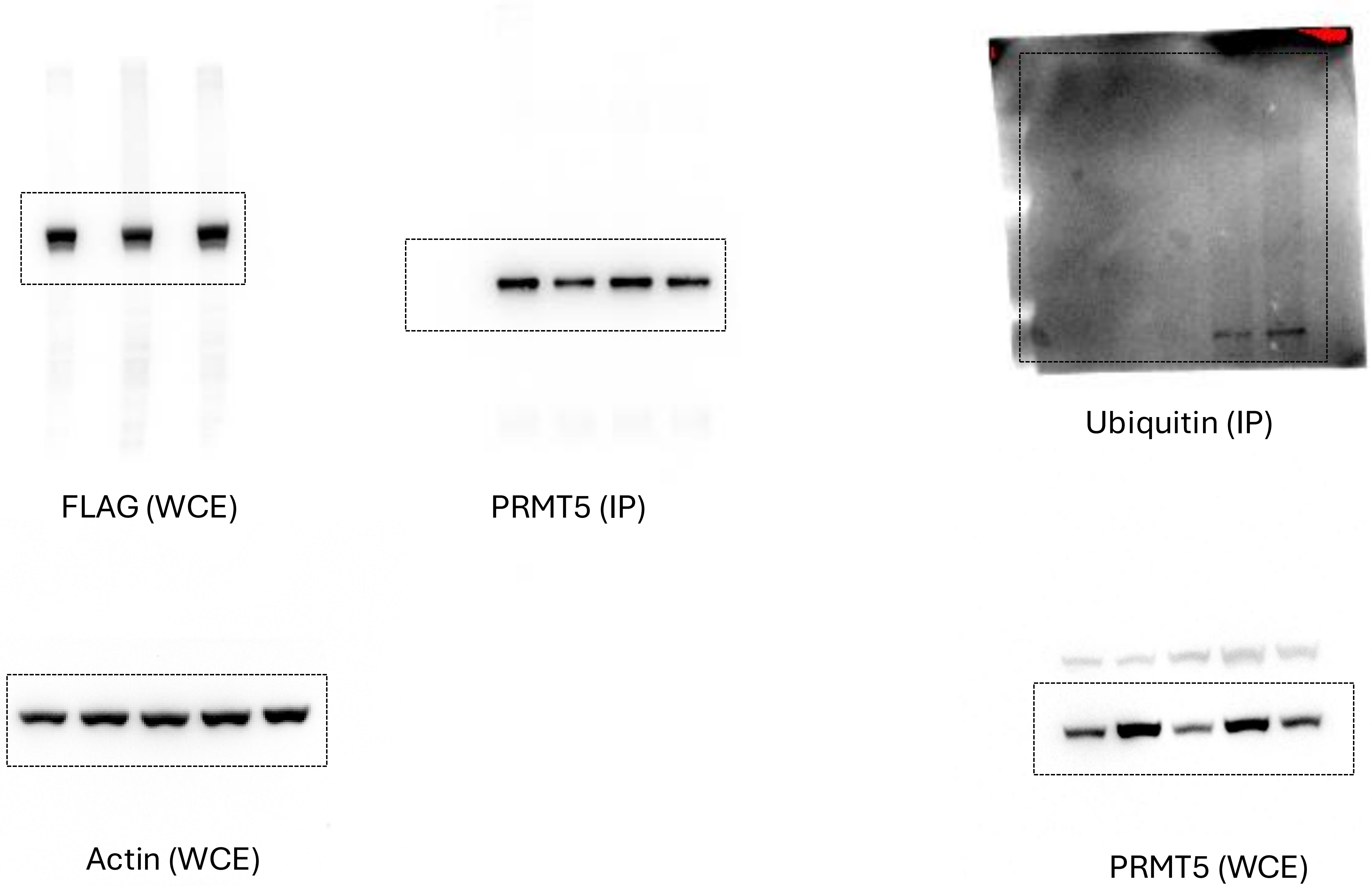

Fig. 5D

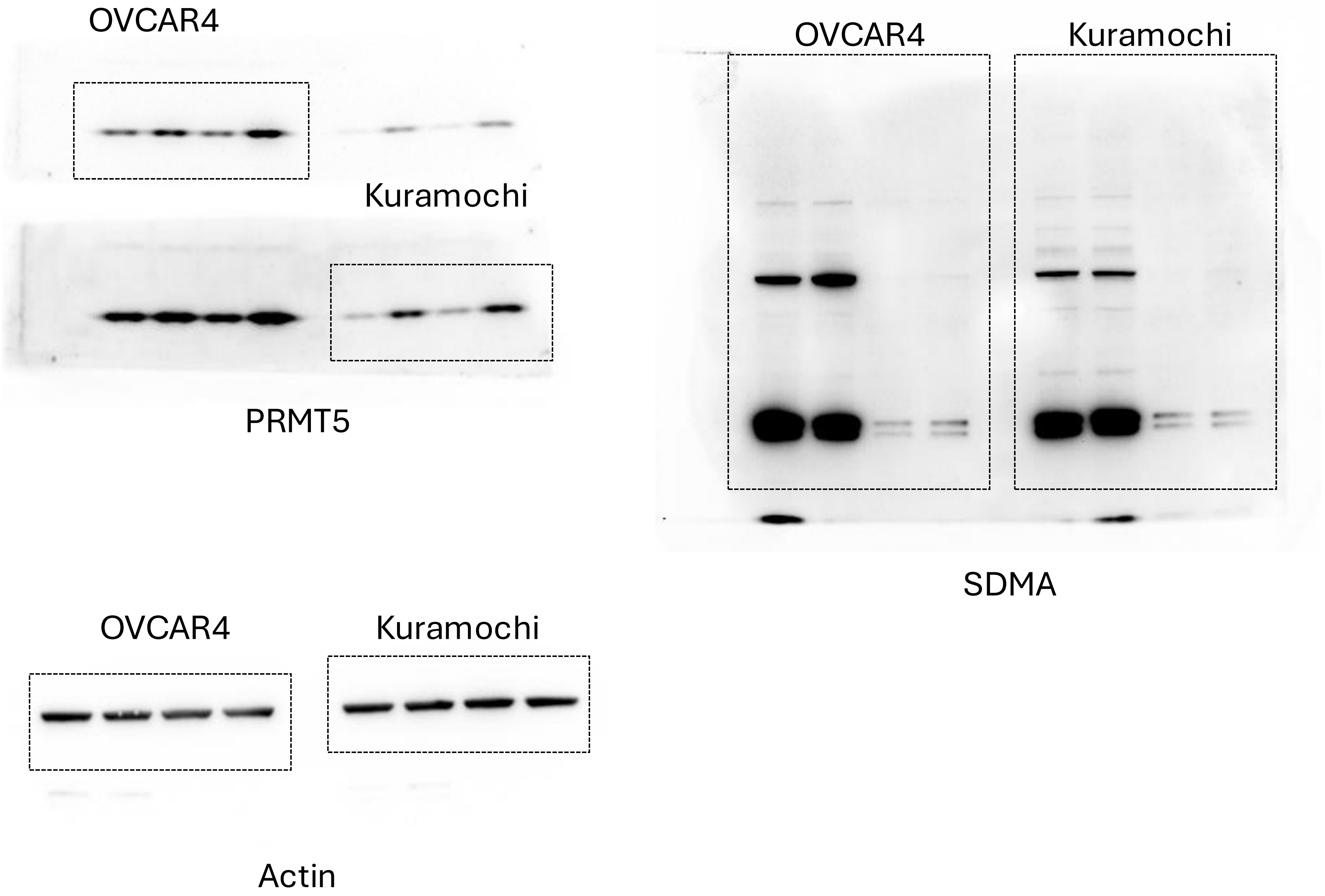
OVCAR4
Kuramochi
PRMT5
OVCAR4
Kuramochi
SDMA
OVCAR4
Kuramochi
Actin

Sup. Fig. 1B

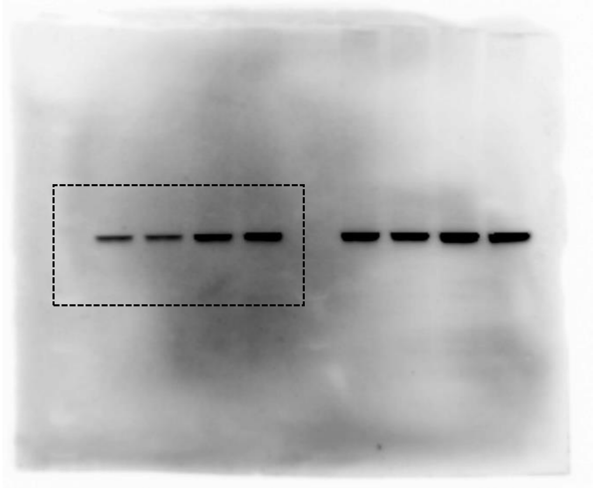

PRMT5

Tp53

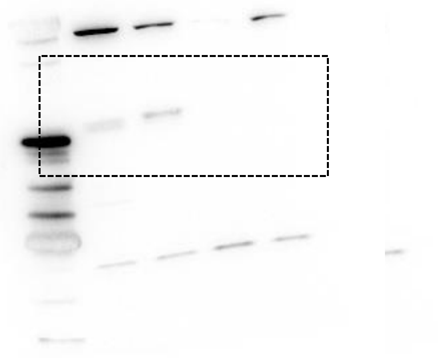

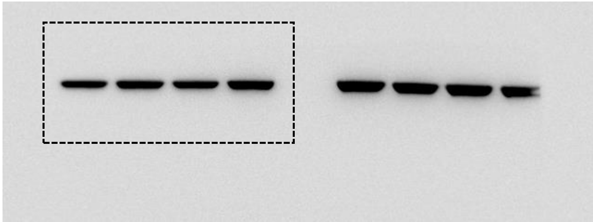

Actin

Sup. Fig. 3D-E

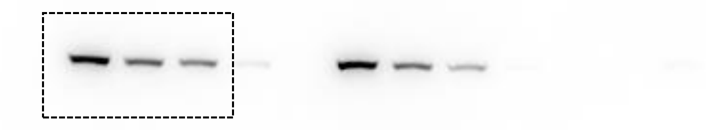

PRMT5

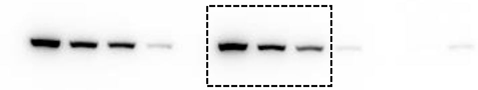

PRMT5

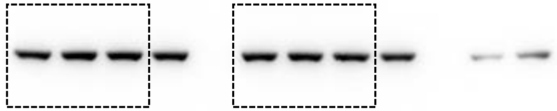

Actin

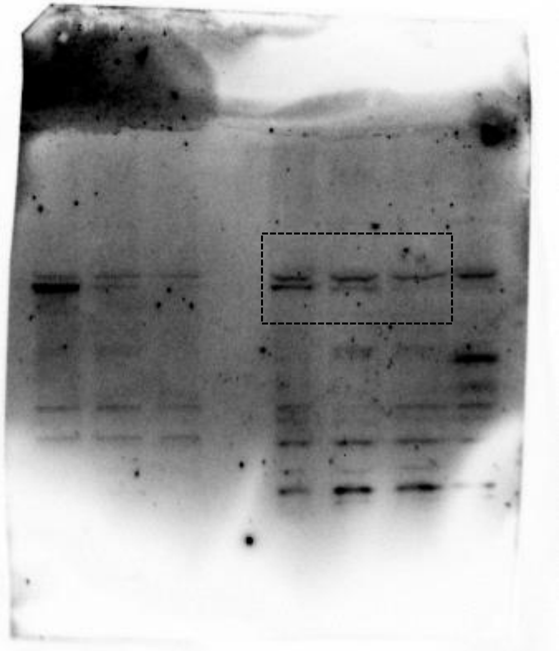

FER

Sup. Fig. 3F

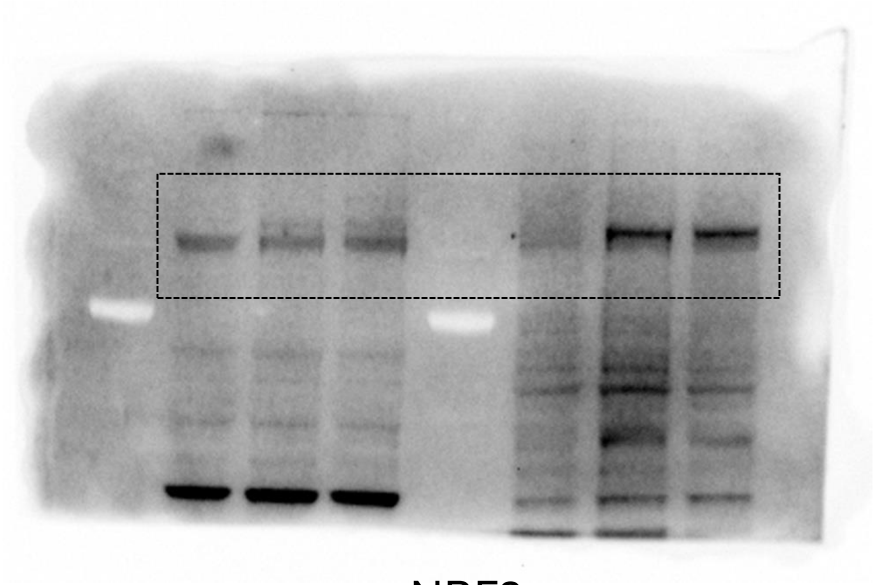

NRF2

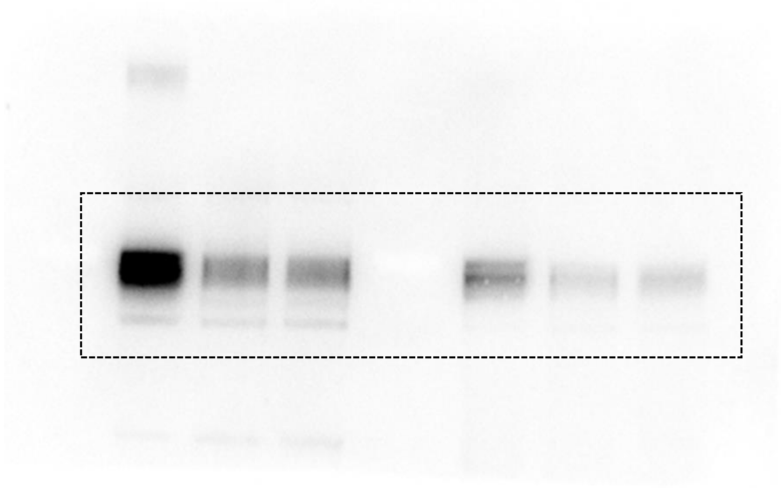

KEAP1

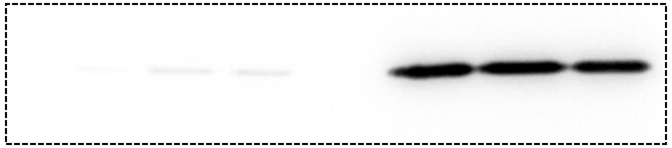

Histone H3

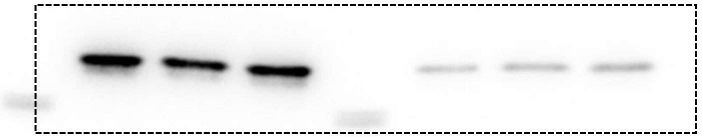

Cyclophilin A

Sup. Fig. 4C

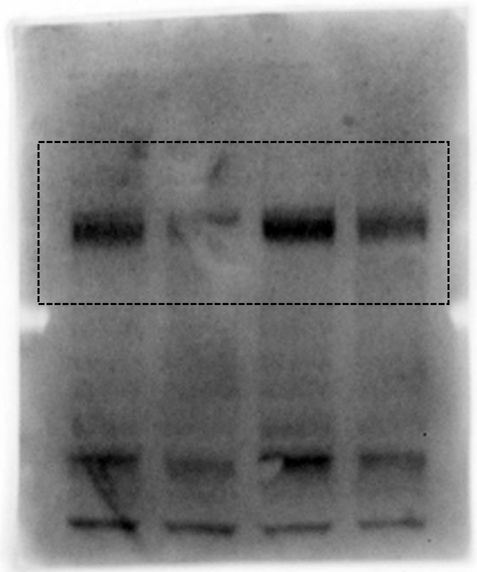

NRF2

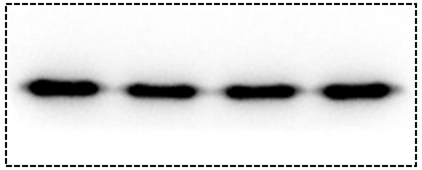

Histone H3
